# Synthesis, activity and mechanism of alkoxy-, carbamato-, sulfonamido-, thioureido-, and ureido-derivatives of 2,4,5-trimethylpyridin-3-ol against inflammatory bowel disease

**DOI:** 10.1080/14756366.2019.1677637

**Published:** 2019-10-16

**Authors:** Chhabi Lal Chaudhary, Pallavi Gurung, Seoul Jang, Suhrid Banskota, Tae-Gyu Nam, Jung-Ae Kim, Byeong-Seon Jeong

**Affiliations:** a College of Pharmacy and Institute for Drug Research, Yeungnam University, Gyeongsan, Republic of Korea; b Department of Pharmacy and Institute of Pharmaceutical Science and Technology, Hanyang University, Ansan, Republic of Korea

**Keywords:** Pyridin-3-ols, inflammatory bowel disease, TNF-α, adhesion, epithelial junction

## Abstract

Inflammatory bowel disease (IBD) is a chronic immuno-inflammation in gastrointestinal tract. We have evaluated the activity of the compounds to inhibit the adhesion of monocytes to colon epithelial cells is triggered by a pro-inflammatory cytokine, tumour necrosis factor (TNF)-α. The *in vitro* activity of the compounds, **13b** (an ureido-derivative), **14c**, **14j, 14k, 14n** (thioureido-), **18c** and **18d** (sulfonamido-), was in correlation with *in vivo* anti-colitis activity revealed as significant recovery in body- and colon-weights and colon myeloperoxidase level, a biochemical marker of inflammation reflecting neutrophil infiltration. *In vivo*, TNBS-induced changes in the expression of inflammatory cytokines (TNF-α, IL-6, IL-1β, IL-10, and TGF-β), NLRP3 inflammasome components (NLRP-3, Caspase-1, and IL-18), and epithelial junction molecules (E-cadherin, claudin2/3, and ZO-1) were blocked and recovered by oral administration of the compounds (1 mg/kg). Compound **14n** which showed the best efficacy can be a promising lead for orally available therapeutics for pathology of IBD.

## 1. Introduction

Inflammatory bowel disease (IBD) refers to a chronic inflammation occurring in the gastrointestinal tract with increasing worldwide incidence and prevalence. Two major subtypes of IBD include ulcerative colitis (UC) and Crohn’s disease (CD) with distinct injury site and disease types. The exact cause of IBD is not fully understood, but mounting evidences suggest that its pathogenesis is associated with close interactions between genetic, immune, and environmental factors, resulting in impairment of intestinal epithelial barrier and dysregulation of inflammatory cytokine homeostasis. While a couple of molecular targets have been studied for treatment of IBD, only a few target-based approaches are successful and there are no well-validated molecular targets known so far. The best examples of successful anti-IBD treatment would be anti-tumour necrosis factor-α (anti-TNF-α) antibody therapies such as infliximab and adalimumab used in clinics, showing mucosal healing in CD^1^. However, some patients do not respond to the drugs, which highlights the necessity of new pharmacological interventions for of IBD. Examples of small molecule inhibitors with good therapeutic outcome are even scarcer. A Janus kinase (JAK) inhibitor, tofacitinib has recently been included as a therapeutic option in the treatment of UC and possibly of CD^2^. Therefore, medical unmet need for small molecule IBD treatment still remains huge.

Uncontrolled inflammatory response results from an imbalance between the levels of proinflammatory cytokines such as TNF-α, IL-6, IL-1β, and anti-inflammatory cytokines like IL-10 and TGF-β^3^^,^^4^. TNF-α is a master cytokine which induces the production of IL-1β and IL-6 through activation of NF-kB, leading to a vicious cycle of persistent inflammation and deterioration of epithelial function^5^^,^^6^. IL-10 and TGF-β which downregulate proinflammatory cytokine (TNF-α and IL-6) production and Th1 immune response, also play a crucial role in maintenance of colon mucosa integrity during inflammation^7–9^. The effects of IL-10 depend on the timing and sources of its secretion. In low to moderate inflammatory condition, IL-10 from dendritic cells or macrophages drives the production of IL-10 by regulatory T cells (Treg), whereas in case of challenging with strong proinflammatory response condition, a large amount of IL-10 is produced from larger populations of cells to minimise pathology during the resolution phase of the challenge^10^.

During cellular stress or infection, an innate immune response occurs through pattern recognition receptors (PRRs) like toll-like receptors (TLR) and NOD-like receptors. NOD-like receptor protein 3 (NLRP3), one of the most characterised PRRs, forms a multiprotein complex called inflammasome, leading to caspase-1 activation and release of IL-1β and IL-18^11^. NLRP3 further triggers pyroptosis, an inflammatory programmed cell death, and thus intestinal barrier damage^12^. Abnormal activation of NLRP3 inflammasome has been linked to IBD^13^. Based on the findings that inhibition of NLRP3 blocks not only cytokine release but also p’yroptotic cell death, NLRP3 inflammasome is proposed to be a potential target in IBD drug discovery efforts^13^.

**Figure 1.**
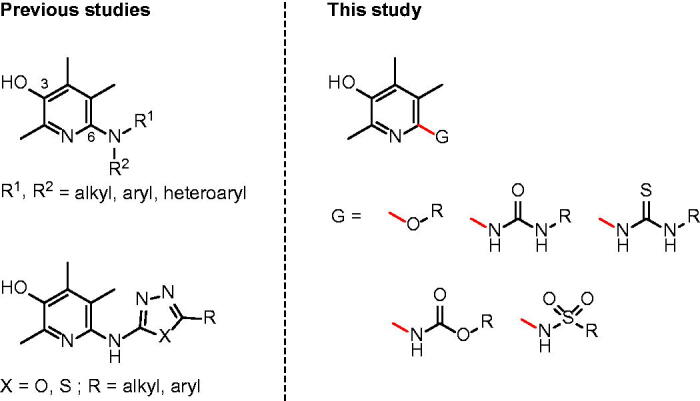
2,4,5-Trimethylpyridin-3-ol as anti-IBD scaffold.

For the last several years, we have conducted a phenotype-based approach to identify small molecule inhibitors^14–19^. Based on the notion that immune cells such as monocytes infiltrate and adhere to colon epithelium in the presence of TNF-α, we have developed a cell-based screening assay to monitor the cell adhesion phenotype in the presence of compounds. We recently published a couple of papers on the discovery of 6-aminopyridin-3-ols to inhibit IBD *in vitro* and *in vivo* (Figure 1). Among the many compounds identified as effective in the previous studies, the analogues having (4-propylphenyl)amino, (6-chloropyridin-3-yl)amino, and (5-phenyl-1,3,4-thiadiazol-2-yl)amino substructures at C(6)-position could be of particular excellence. These three compounds showed >75% inhibitory effect against TNF-α-induced cell adhesion between monocyte and colon epithelial cells at 1 μM concentration. Considering that 5-aminosalicylic acid (5-ASA, mesalazine), an active metabolite of sulfasalazine (SSZ) which is widely used to treat IBD in the clinical field, has only 3.5% inhibitory activity at the same drug concentration (1 μM), the *in vitro* activity of our three compounds can be quite marvellous. Moreover, *in vivo* efficacy studies using rats with severe colon inflammation induced by 2,4,6-trinitrobenzenesulfonic acid (TNBS) have confirmed that our compounds are certainly effective against IBD. When orally administered at the dose of 1 mg/kg, those compounds showed very good efficacy demonstrated by ameliorating disease parameters such as % of the recovery in colon- and body-weights (up to 79% and 59%, respectively) and myeloperoxidase (MPO) level.

In this study, we made some changes in the functional group at C(6)-position of the pyridin-3-ol ring and examined how these structural changes affect on efficacy against IBD. We considered five types of functional groups (alkoxy-, ureido-, thioureido-, carbamato-, and sulfonamido-group) to replace 6-amino groups, and synthesised several to a dozen new derivatives for each family (Figure 1).

## 2. Materials and methods

### 2.1. Chemistry

Unless noted otherwise, materials were purchased from commercial suppliers and used without further purification. Air- or moisture-sensitive reactions were carried out under an inert gas atmosphere. Reaction progress was monitored by thin-layer-chromatography (TLC) using silica gel F_254_ plates. The products were purified by flash column chromatography using silica gel 60 (70–230 mesh) or by Biotage ‘Isolera One’ system with indicated solvents. Melting points were determined using a Fischer-Jones melting point apparatus and were not corrected. NMR spectra were obtained using Bruker spectrometers 250 MHz or 400 MHz for ^1^H-NMR and 62.5 MHz or 100 MHz for ^13 ^C-NMR. Chemical shifts (δ) were expressed in ppm using solvent as an internal standard and coupling constant (*J*) in hertz. Low-resolution mass spectra (LRMS) were obtained using an Advion Expression CMS, and recorded in a positive ion mode with an electrospray (ESI) source. Described below are detailed methods for synthesising the new compounds introduced in this paper. Other compounds were prepared using the synthetic methods we had previously reported^20^.

#### 2.1.1. 5-(Benzyloxy)-3,4,6-trimethylpyridin-2-ol (6)

To a solution of 5-benzyloxy-3,4,6-trimethylpyridin-6-amine (**5**, 2 g, 8.25 mmol) in a mixed solvent of THF (3 ml) and water (100 ml) was added 10% H_2_SO_4_ (1.1 ml, 20.63 mmol) followed by addition of NaNO_2_ (285 mg, 4.13 mmol) at 0 °C. The reaction temperature was gradually increased to room temperature and stirred for 2 h. The reaction mixture was diluted with CH_2_Cl_2_; washed with sat. NaHCO_3_. The aqueous layer separated was extracted with CH_2_Cl_2_. The combined CH_2_Cl_2_ solution was washed with brine, dried over MgSO_4_, filtered, and concentrated to give **6** (1.95 g, 98%). Yellow solid; TLC R_f_ 0.20 (CH_2_Cl_2_/MeOH = 20/1); m.p. 198 °C; MS (ESI) *m/z* 244 [M + H]^+^; ^1^H-NMR (CDCl_3_) *δ* 13.04 (s, 1H), 7.48–7.32 (m, 5H), 4.70 (s, 2H), 2.28 (s, 3H), 2.17 (s, 3H), 2.10 (s, 3H); ^13 ^C-NMR (CDCl_3_) *δ* 163.20, 145.35, 139.06, 136.95, 133.43, 128.75 (2 C), 128.42, 128.20 (2 C), 123.53, 75.83, 13.98, 13.85, 12.49.

#### 2.1.2. 2,5-Bis(benzyloxy)-3,4,6-trimethylpyridine (7a)

To a solution of 5-(benzyloxy)-3,4,6-trimethylpyridin-2-ol (**6**, 50 mg, 0.21 mmol) in a mixed solvent of DMF (1 ml) and THF (1 ml) was added Ag_2_CO_3_ (69 mg, 0.25 mmol) followed by addition of 3-bromo-1-propylbenzeze (38 µL, 0.32 mmol). The mixture was stirred at room temperature for 24 h. After filtration of the reaction mixture through a Celite pad, the filtrate was diluted with CH_2_Cl_2_ and water. The aqueous layer separated was extracted with CH_2_Cl_2_, and the combined CH_2_Cl_2_ solution was dried over MgSO_4_, filtered and concentrated. The residue was purified by silica gel column chromatography (Hexanes/EtOAc = 20/1) to give **7a** (60 mg, 88%). White solid; TLC R_f_ 0.61 (Hexanes/EtOAc = 10/1); m.p. 64 °C; MS (ESI) *m/z* 334 [M + H]^+^; ^1^H-NMR (CDCl_3_) *δ* 7.54–7.29 (m, 10H), 5.39 (s, 2H), 4.76 (s, 2H), 2.44 (s, 3H), 2.22 (s, 3H), 2.16 (s, 3H); ^13 ^C-NMR (CDCl_3_) *δ* 156.88, 146.70, 144.54, 141.09, 138.43, 137.33, 128.57 (2 C), 128.28 (2 C), 128.10, 127.92 (2 C), 127.68 (2 C), 127.39, 117.07, 74.97, 67.26, 18.97, 12.78, 11.83.

#### 2.1.3. 3,4,6-Trimethylpyridine-2,5-diol (8a)

To a suspension of 10% palladium on activated carbon (5 mg) in MeOH (2 ml) was added **7a** (20 mg, 0.06 mmol). The mixture was stirred with hydrogen balloon at room temperature for 1 h. After filtration of the reaction mixture through a Celite pad, the filtrate was concentrated. The residue was purified by silica gel column chromatography (CH_2_Cl_2_/MeOH = 20/1) to give **8a** (9 mg, 98%). White solid; TLC R_f_ 0.27 (CH_2_Cl_2_/MeOH = 9/1); m.p. 176 °C; MS (ESI) *m/z* 154 [M + H]^+^; ^1^H-NMR (DMSO-*d_6_*) *δ* 10.96 (s, 1H), 7.51 (s, 1H), 2.07 (s, 3H), 2.03 (s, 3H), 1.90 (s, 3H); ^13 ^C-NMR (DMSO-*d_6_*) *δ* 160.04, 141.97, 134.45, 126.76, 121.30, 13.70, 13.55, 12.27.

#### 2.1.4. 3-(Benzyloxy)-6-butoxy-2,4,5-trimethylpyridine (7 b)

To a solution of 5-(benzyloxy)-3,4,6-trimethylpyridin-2-ol (**6**, 100 mg, 0.41 mmol) in DMF (4 ml) was added Ag_2_CO_3_ (136 mg, 0.49 mmol) followed by addition of 1-iodobutane (70 µL, 0.62 mmol). The mixture was stirred at 40 °C for 2 h. After filtration of the reaction mixture through a Celite pad, the filtrate was concentrated then the residue was diluted with EtOAc and washed with water and brine. The EtOAc solution was dried over MgSO_4_, filtered and concentrated. The residue was further purified by silica gel column chromatography (Hexanes/EtOAc = 30/1) to give **7 b** (87 mg, 71%). Pale yellow solid; TLC R_f_ 0.29 (Hexanes/EtOAc = 20/1); m.p. 33 °C; MS (ESI) *m/z* 300 [M + H]^+^; ^1^H-NMR (CDCl_3_) *δ* 7.55–7.31 (m, 5H), 4.74 (s, 2H), 4.28 (t, *J* = 6.5 Hz, 2H), 2.41 (s, 3H), 2.20 (s, 3H), 2.11 (s, 3H), 1.76 (dt, *J* = 14.6, 6.5 Hz, 2H), 1.51 (dq, *J* = 14.2, 7.3 Hz, 2H), 0.99 (t, *J* = 7.3 Hz, 3H); ^13 ^C-NMR (CDCl_3_) *δ* 157.57, 146.47, 144.59, 140.90, 137.53, 128.69 (2 C), 128.21, 128.06 (2 C), 117.08, 75.09, 65.57, 31.58, 19.62, 19.12, 14.12, 12.87, 11.87.

#### 2.1.5. 6-Butoxy-2,4,5-trimethylpyridin-3-ol (8 b)

To a suspension of 10% palladium on activated carbon (11 mg) in MeOH (2 ml) was added **7 b** (57 mg, 0.19 mmol). The mixture was stirred with hydrogen balloon at room temperature for 1 h. After filtration of the reaction mixture through a Celite pad, the filtrate was concentrated. The residue was dissolved in MeOH and filtered through a membrane filter. The filtrate was concentrated to give **8 b** (28 mg, 70%). Yellow solid; TLC R_f_ 0.41 (CH_2_Cl_2_/MeOH = 20/1); m.p. 63 °C; MS (ESI) *m/z* 210 [M + H]^+^; ^1^H-NMR (CDCl_3_) *δ* 4.23 (t, *J* = 6.5 Hz, 2H), 4.07 (s, 1H), 2.35 (s, 3H), 2.16 (s, 3H), 2.10 (s, 3H), 1.79–1.65 (m, 2H), 1.47 (dd, *J* = 15.0, 7.5 Hz, 2H), 0.97 (t, *J* = 7.3 Hz, 3H); ^13 ^C-NMR (CDCl_3_) *δ* 155.76, 143.03, 136.41, 134.63, 117.05, 65.54, 31.62, 19.61, 18.61, 14.12, 12.25, 11.86.

#### 2.1.6. 3-(Benzyloxy)-2,4,5-trimethyl-6-(octyloxy)pyridine (7c)

To a solution of 5-(benzyloxy)-3,4,6-trimethylpyridin-2-ol (**6**, 100 mg, 0.41 mmol) in DMF (4 ml) was added Ag_2_CO_3_ (136 mg, 0.49 mmol) followed by addition of 1-iodooctane (111 µL, 0.62 mmol). The mixture was stirred at 40 °C for 4 h. After filtration of the reaction mixture through a Celite pad, the filtrate was concentrated. The residue was diluted with EtOAc and washed with water and brine. The EtOAc solution was dried over MgSO_4_, filtered and concentrated. The residue was purified by silica gel column chromatography (Hexanes/EtOAc = 30/1) to give **7c** (131 mg, 90%). Pale yellow solid; TLC R_f_ 0.28 (Hexanes/EtOAc = 20/1); m.p. 31 °C; MS (ESI) *m/z* 356 [M + H]^+^; ^1^H-NMR (CDCl_3_) *δ* 7.52–7.32 (m, 5H), 4.73 (s, 2H), 4.27 (t, *J* = 6.6 Hz, 2H), 2.40 (s, 3H), 2.19 (s, 3H), 2.10 (s, 3H), 1.75 (dd, *J* = 14.3, 6.6 Hz, 2H), 1.32 (m, 10H), 0.89 (t, *J* = 6.5 Hz, 3H); ^13 ^C-NMR (CDCl_3_) *δ* 157.58, 146.54, 144.60, 140.99, 137.58, 128.71 (2 C), 128.22, 128.08 (2 C), 117.15, 75.14, 65.99, 32.02, 29.58, 29.45 (2 C), 26.42, 22.83, 19.10, 14.24, 12.90, 11.89.

#### 2.1.7. 2,4,5-Trimethyl-6-(octyloxy)pyridin-3-ol (8c)

To a suspension of 10% palladium on activated carbon (20 mg) in MeOH (2 ml) was added **7c** (99 mg, 0.28 mmol). The mixture was stirred with hydrogen balloon at room temperature for 1 h. After filtration of the reaction mixture through a Celite pad, the filtrate was concentrated. The residue was dissolved in MeOH and filtered through a membrane filter. The filtrate was concentrated to give **8c** (70 mg, 95%). Pale yellow solid; TLC R_f_ 0.48 (CH_2_Cl_2_/MeOH = 20/1); m.p. 52 °C; MS (ESI) *m/z* 266 [M + H]^+^; ^1^H-NMR (CDCl_3_) *δ* 4.22 (t, *J* = 6.6 Hz, 2H), 4.14 (s, 1H), 2.35 (s, 3H), 2.16 (s, 3H), 2.10 (s, 3H), 1.72 (dd, *J* = 14.2, 6.7 Hz, 2H), 1.51–1.18 (m, 10H), 0.89 (dd, *J* = 8.9, 4.4 Hz, 3H); ^13 ^C-NMR (CDCl_3_) *δ* 155.75, 143.05, 136.47, 134.77, 117.09, 65.93, 32.00, 29.57, 29.46, 29.44, 26.40, 22.83, 18.58, 14.25, 12.27, 11.87.

#### 2.1.8. 2,4,5-Trimethyl-6-(octyloxy)pyridin-3-ol (7d)

To a solution of 5-(benzyloxy)-3,4,6-trimethylpyridin-2-ol (**6**, 100 mg, 0.41 mmol) in DMF (4 ml) was added Ag_2_CO_3_ (136 mg, 0.49 mmol) followed by addition of 1-iodo-3-methylbutane (71 µL, 0.62 mmol). The mixture was stirred at 50 °C for 20 h. After filtration of the reaction mixture through a Celite pad, the filtrate was concentrated. The residue was diluted with EtOAc and washed with water and brine. The EtOAc solution was dried over MgSO_4_, filtered and concentrated. The residue was purified by silica gel column chromatography (Hexanes/EtOAc = 30/1) to give **7d** (98 mg, 76%). Colourless liquid; TLC R_f_ 0.30 (Hexanes/EtOAc = 20/1); MS (ESI) *m/z* 514 [M + H]^+^; ^1^H-NMR (CDCl_3_) *δ* 7.52–7.33 (m, 5H), 4.72 (s, 2H), 4.29 (t, *J* = 6.6 Hz, 2H), 2.40 (s, 3H), 2.19 (s, 3H), 2.09 (s, 3H), 1.82 (td, *J* = 13.3, 6.6 Hz, 1H), 1.65 (q, *J* = 6.7 Hz, 2H), 0.97 (d, *J* = 6.6 Hz, 6H); ^13 ^C-NMR (CDCl_3_) *δ* 157.52, 146.42, 144.55, 142.42, 137.46, 128.72 (2 C), 128.24, 128.07 (2 C), 117.11, 75.09, 64.33, 38.26, 25.42, 22.88 (2 C), 19.10, 12.91, 11.92.

#### 2.1.9. 6-(Isopentyloxy)-2,4,5-trimethylpyridin-3-ol (8d)

To a suspension of 10% palladium on activated carbon (14 mg) in MeOH (2 ml) was added **7d** (72 mg, 0.23 mmol). The mixture was stirred with hydrogen balloon at room temperature for 1 h. After filtration of the reaction mixture through a Celite pad, the filtrate was concentrated. The residue was dissolved in MeOH and filtered through a membrane filter. The filtrate was concentrated to give **8d** (40 mg, 78%). Pale yellow liquid; TLC R_f_ 0.41 (CH_2_Cl_2_/MeOH = 20/1); MS (ESI) *m/z* 224 [M + H]^+^; ^1^H-NMR (CDCl_3_) *δ* 4.26 (t, *J* = 6.6 Hz, 2H), 4.10 (s, 1H), 2.36 (s, 3H), 2.16 (s, 3H), 2.09 (s, 3H), 1.81 (dt, *J* = 13.3, 6.7 Hz, 1H), 1.63 (q, *J* = 6.7 Hz, 2H), 0.96 (d, *J* = 6.6 Hz, 6H); ^13 ^C-NMR (CDCl_3_) *δ* 155.72, 143.05, 136.40, 134.75, 117.08, 64.33, 38.34, 25.46, 22.87 (2 C), 18.58, 12.27, 11.88.

#### 2.1.10. 6-(Isopentyloxy)-2,4,5-trimethylpyridin-3-ol (7e)

To a solution of 5-(benzyloxy)-3,4,6-trimethylpyridin-2-ol (**6**, 100 mg, 0.41 mmol) in DMF (4 ml) was added Ag_2_CO_3_ (136 mg, 0.49 mmol) followed by addition of 1-iodocyclopentane (71 µL, 0.62 mmol). The mixture was stirred at 50 °C for 7 h. After filtration of the reaction mixture through a Celite pad, the filtrate was concentrated. The residue was diluted with EtOAc and washed with water and brine. The EtOAc solution was dried over MgSO_4_, filtered and concentrated. The residue was purified by silica gel column chromatography (Hexanes/EtOAc = 50/1) to give **7e** (88 mg, 69%). Colourless liquid; TLC R_f_ 0.29 (Hexanes/EtOAc = 20/1); MS (ESI) *m/z* 312 [M + H]^+^; ^1^H-NMR (CDCl_3_) *δ* 7.52–7.31 (m, 5H), 5.54–5.39 (m, 1H), 4.74 (s, 2H), 2.39 (s, 3H), 2.18 (s, 3H), 2.06 (s, 3H), 1.93 (dd, *J* = 10.8, 6.3 Hz, 2H), 1.77 (dd, *J* = 20.6, 5.4 Hz, 4H), 1.63 (dd, *J* = 10.9, 4.1 Hz, 2H); ^13 ^C-NMR (CDCl_3_) *δ* 157.18, 146.30, 144.66, 140.72, 137.60, 128.69 (2 C), 128.19, 128.05 (2 C), 117.49, 75.07, 33.09 (2 C), 29.86, 24.07 (2 C), 19.26, 12.89, 11.9.

#### 2.1.11. 6-(Cyclopentyloxy)-2,4,5-trimethylpyridin-3-ol (8e)

To a suspension of 10% palladium on activated carbon (13 mg) in MeOH (2 ml) was added **7e** (63 mg, 0.20 mmol). The mixture was stirred with hydrogen balloon at room temperature for 1 h. After filtration of the reaction mixture through a Celite pad, the filtrate was concentrated. The residue was dissolved in MeOH and filtered through a membrane filter. The filtrate was concentrated to give **8e** (39 mg, 87%). Pale yellow solid; TLC R_f_ 0.41 (CH_2_Cl_2_/MeOH = 20/1); m.p. 79 °C; MS (ESI) *m/z* 222 [M + H]^+^; ^1^H-NMR (CDCl_3_) *δ* 5.38 (dt, *J* = 8.2, 3.0 Hz, 1H), 4.18 (s, 1H), 2.34 (s, 3H), 2.14 (s, 3H), 2.06 (s, 3H), 1.95–1.82 (m, 2H), 1.82–1.67 (m, 4H), 1.66–1.51 (m, 2H); ^13 ^C-NMR (CDCl_3_) *δ* 155.33, 142.90, 136.71, 134.56, 117.61, 77.46, 33.03 (2 C), 24.03 (2 C), 18.71, 12.26, 11.92.

#### 2.1.12. 3-(Benzyloxy)-2,4,5-trimethyl-6–(3-phenylpropoxy)pyridine (7f)

To a solution of 5-(benzyloxy)-3,4,6-trimethylpyridin-2-ol (**6**, 30 mg, 0.12 mmol) in DMF (1 ml) was added Ag_2_CO_3_ (41 mg, 0.15 mmol) followed by addition of 3-bromo-1-propylbenzeze (28 µL, 0.19 mmol). The mixture was stirred at 80 °C for 12 h. After filtration of the reaction mixture through a Celite pad, the filtrate was concentrated. The residue was diluted with EtOAc and washed with water and brine. The EtOAc solution was dried over MgSO_4_, filtered and concentrated. The residue was purified by silica gel column chromatography (Hexanes/EtOAc = 30/1) to give **7f** (37 mg, 83%). Colourless liquid; TLC R_f_ 0.31 (Hexanes/EtOAc = 15/1); MS (ESI) *m/z* 362 [M + H]^+^; ^1^H-NMR (CDCl_3_) *δ* 7.54–7.36 (m, 5H), 7.36–7.17 (m, 5H), 4.76 (s, 2H), 4.35 (t, *J* = 6.3 Hz, 2H), 2.84 (dd, *J* = 8.6, 6.9 Hz, 2H), 2.42 (s, 3H), 2.23 (s, 3H), 2.15 (s, 3H), 2.14–2.05 (m, 2H); ^13 ^C-NMR (CDCl_3_) *δ* 157.35, 146.57, 144.65, 142.21, 140.96, 137.51, 128.69 (2 C), 128.62 (2 C), 128.46 (2 C), 128.21, 128.05 (2 C), 125.88, 117.04, 75.08, 64.95, 32.66, 31.09, 19.12, 12.88, 11.89.

#### 2.1.13. 2,4,5-Trimethyl-6–(3-phenylpropoxy)pyridin-3-ol (8f)

To a suspension of 10% palladium on activated carbon (15 mg) in MeOH (2 ml) was added **7f** (77 mg, 0.21 mmol). The mixture was stirred with hydrogen balloon at room temperature for 1 h. After filtration of the reaction mixture through a Celite pad, the filtrate was concentrated. The residue was dissolved in MeOH and filtered through a membrane filter. The filtrate was concentrated to give **8f** (50 mg, 87%). White solid; TLC R_f_ 0.30 (CH_2_Cl_2_/MeOH = 20/1); m.p. 73 °C; MS (ESI) *m/z* 272 [M + H]^+^; ^1^H-NMR (CDCl_3_) *δ* 7.35–7.16 (m, 5H), 4.29 (t, *J* = 6.3 Hz, 3H), 2.89–2.71 (m, 2H), 2.36 (s, 3H), 2.18 (s, 3H), 2.14 (s, 3H), 2.13–2.02 (m, 2H); ^13 ^C-NMR (CDCl_3_) *δ* 155.55, 143.15, 142.24, 136.65, 134.92, 128.61 (2 C), 128.44 (2 C), 125.86, 117.04, 65.00, 32.64, 31.14, 18.55, 12.28, 11.87.

#### 2.1.14. 1–(5-(Benzyloxy)-3,4,6-trimethylpyridin-2-yl)-3-propylurea (11a)

To a solution of 5-(benzyloxy)-3,4,6-trimethylpyridin-6-amine (**5**, 100 mg, 0.41 mmol) in CH_2_Cl_2_ (5 ml) was added propyl isocyanate (46 μL, 0.50 mmol). The mixture was stirred at room temperature for 40 h. After concentration of the reaction mixture, the residue was purified by silica gel column chromatography (CHCl_3_/MeOH = 50/1 to 20/1) to give **13a** (125 mg, 92%). White solid; TLC R_f_ 0.34 (CH_2_Cl_2_/MeOH = 30/1); m.p. 175 °C; MS (ESI) *m/z* 328 [M + H]^+^; ^1^H-NMR (CDCl_3_) *δ* 9.79 (s, 1H), 7.49–7.33 (m, 5H), 6.69 (s, 1H), 4.74 (s, 2H), 3.35 (td, *J* = 6.9, 5.6 Hz, 2H), 2.40 (s, 3H), 2.22 (s, 3H), 2.10 (s, 3H), 1.64 (dd, *J* = 14.3, 7.1 Hz, 2H), 1.02 (t, *J* = 7.4 Hz, 3H); ^13 ^C-NMR (CDCl_3_) *δ* 155.95, 146.87, 146.75, 144.92, 141.54, 137.01, 128.77 (2 C), 128.43, 128.10 (2 C), 115.89, 75.38, 41.73, 23.20, 19.11, 13.31, 12.84, 11.75.

#### 2.1.15. 1–(5-Hydroxy-3,4,6-trimethylpyridin-2-yl)-3-propylurea (13a)

To a suspension of 10% palladium on activated carbon (21 mg) in CH_2_Cl_2_ (4 ml) was added **11a** (105 mg, 0.32 mmol). The mixture was stirred with hydrogen balloon at room temperature for 2 h. After filtration of the reaction mixture through a Celite pad, the filtrate was concentrated. The residue was purified by silica gel column chromatography (CH_2_Cl_2_/MeOH = 20/1) to give **13a** (58 mg, 75%). White solid; TLC R_f_ 0.26 (CH_2_Cl_2_/MeOH = 20/1); m.p. 176 °C; MS (ESI) *m/z* 239 [M + H]^+^; ^1^H-NMR (DMSO-*d_6_*) *δ* 8.88 (s, 1H), 8.20 (s, 1H), 7.71 (s, 1H), 3.14 (dd, *J* = 12.4, 6.7 Hz, 2H), 2.29 (s, 3H), 2.12 (s, 3H), 2.05 (s, 3H), 1.56–1.41 (m, 2H), 0.91 (t, *J* = 7.4 Hz, 3H); ^13 ^C-NMR (DMSO-*d_6_*) *δ* 155.50, 144.40, 143.36, 138.73, 135.53, 118.36, 40.75, 22.78, 19.13, 13.08, 12.66, 11.39.

#### 2.1.16. 1–(5-(Benzyloxy)-3,4,6-trimethylpyridin-2-yl)-3–(4-nitrophenyl)urea (11k)

To a solution of 5-(benzyloxy)-3,4,6-trimethylpyridin-6-amine (**5**, 50 mg, 0.21 mmol) in CH_2_Cl_2_ (2 ml) was added 4-nitrophenyl isocyanate (41 mg, 0.25 mmol). The mixture was stirred at room temperature for 4 h. The reaction mixture was diluted Et_2_O and the precipitate was filtered. The filter cake was washed with Et_2_O and acetone to give **11k** (73 mg, 87%). White solid; TLC R_f_ 0.33 (CH_2_Cl_2_/MeOH = 40/1); m.p. 227 °C; MS (ESI) *m/z* 429 [M + Na]^+^; ^1^H-NMR (DMSO-*d_6_*) *δ* 11.77 (s, 1H), 8.72 (s, 1H), 8.21 (d, *J* = 8.4 Hz, 2H), 7.77 (d, *J* = 8.5 Hz, 2H), 7.60–7.32 (m, 5H), 4.81 (s, 2H), 2.44 (s, 3H), 2.22 (s, 3H), 2.16 (s, 3H); ^13 ^C-NMR (DMSO-*d_6_*) *δ* 152.21, 147.72, 145.66, 145.12, 144.96, 141.49, 141.38, 136.80, 128.22 (2 C), 127.98 (2 C), 127.90, 124.87 (2 C), 120.88, 118.06 (2 C), 74.34, 18.59, 13.18, 12.75.

#### 2.1.17. 1–(4-Aminophenyl)-3–(5-hydroxy-3,4,6-trimethylpyridin-2-yl)urea (13k)

To a suspension of 10% palladium on activated carbon (15 mg) in MeOH (3 ml) was added **11k** (75 mg, 0.18 mmol). The mixture was stirred with hydrogen balloon at room temperature for 12 h. After filtration of the reaction mixture through a Celite pad, the filtrate was concentrated. The residue was purified by silica gel column chromatography (CH_2_Cl_2_/MeOH = 20/1) to give **13k** (14 mg, 26%). White solid; TLC R_f_ 0.35 (CH_2_Cl_2_/MeOH = 20/1); m.p. 200 °C (decomp.); MS (ESI) *m/z* 309 [M + Na]^+^; ^1^H-NMR (DMSO-*d_6_*) *δ* 11.03 (s, 1H), 8.34 (s, 1H), 7.94 (s, 1H), 7.15 (d, *J* = 8.7 Hz, 2H), 6.52 (d, *J* = 8.7 Hz, 2H), 4.77 (s, 2H), 2.36 (s, 3H), 2.14 (s, 3H), 2.10 (s, 3H); ^13 ^C-NMR (DMSO-*d_6_*) *δ* 153.01, 144.74, 144.09, 142.99, 138.81, 135.83, 128.50, 120.61 (2 C), 118.97, 114.17 (2 C), 19.20, 13.19, 12.76.

#### 2.1.18. 1–(5-(Benzyloxy)-3,4,6-trimethylpyridin-2-yl)thiourea (12a)

To a solution of *N*-((5-(benzyloxy)-3,4,6-trimethylpyridin-2-yl)carbamothioyl)benzamide (**12n**, 100 mg, 0.25 mmol) in a mixed solvent of THF (1 ml) and water (1 ml) was added NaOH (20 mg, 0.49 mmol). The mixture was refluxed for 17 h. The reaction mixture was cooled to room temperature and diluted with water. The pH of the reaction mixture was adjusted to around 7 using 2 M HCl. The precipitate formed was collected by filtration and the filter cake was washed with water to give **12a** (43 mg, 58%). Ivory solid; TLC R_f_ 0.22 (Hex/EtOAc = 2/1); m.p. 165 °C; MS (ESI) *m/z* 302 [M + H]^+^; ^1^H-NMR (CDCl_3_) *δ* 11.26 (s, 1H), 7.96 (s, 1H), 7.46 – 7.35 (m, 5H), 6.84 (s, 1H), 4.76 (s, 2H), 2.40 (s, 3H), 2.24 (s, 3H), 2.18 (s, 3H); ^13 ^C-NMR (CDCl_3_) *δ* 148.10, 148.00, 146.49, 145.99, 142.17, 136.69, 128.83 (2 C), 128.58, 128.14 (2 C), 116.92, 75.41, 19.25, 13.43, 12.99.

#### 2.1.19. 1–(5-Hydroxy-3,4,6-trimethylpyridin-2-yl)thiourea (14a)

To a solution of **12a** (34 mg, 0.11 mmol) in CH_2_Cl_2_ (1 ml), was added pentamethylbenzene (50 mg, 0.34 mmol) followed by dropwise addition of boron trichloride (1 M in CH_2_Cl_2_, 0.2 ml) at 0 °C. The reaction mixture was stirred at 0 °C for 15 min then quenched with CHCl_3_/MeOH solution (9/1, 1.5 ml) and stirred at room temperature for 30 min. After concentration of the reaction mixture, 2 ml of a mixed solvent (CH_2_Cl_2_/MeOH = 40/1) was added to the residue and precipitate formed was filtered, washed with CH_2_Cl_2_ and collected to give **14a** (23.6 mg, 99%). White solid; TLC Rf 0.35 (CH_2_Cl_2_/MeOH = 9/1); m.p. 189 °C; MS (ESI) *m/z* 212 [M + H]^+^; ^1^H-NMR (CD_3_OD) *δ* 2.35 (s, 3H), 2.22 (s, 3H), 2.18 (s, 3H); ^13 ^C-NMR (CD_3_OD) *δ* 147.61, 144.33, 141.39, 137.55, 129.24, 128.91, 19.08, 12.98, 12.77.

#### 2.1.20. Methyl (5-benzyloxy-3,4,6-trimethylpyridin-2-yl)carbamate (15a)

To a solution of 5-benzyloxy-3,4,6-trimethylpyridin-6-amine (**5**, 70 mg, 0.29 mmol) in acetone (1 ml) was added K_2_CO_3_ (120 mg, 0.87 mmol) followed by dropwise addition of methyl chloroformate (112 µL, 1.45 mmol) at 0 °C. The mixture was stirred at room temperature for 24 h. The reaction mixture was concentrated and then diluted with CH_2_Cl_2_ and water. The aqueous layer separated was extracted with CH_2_Cl_2_. The combined CH_2_Cl_2_ solution was washed with brine and dried over MgSO_4_, filtered and concentrated. The residue was purified by silica gel column chromatography (CH_2_Cl_2_/MeOH = 30/1 to 20/1) to give **15a** (63 mg, 73%). Pale yellow solid; TLC R_f_ 0.29 (CH_2_Cl_2_/MeOH = 15/1); m.p. 117 °C; MS (ESI) *m/z* 301 [M + H]^+^; ^1^H-NMR (CDCl_3_) *δ* 7.50–7.32 (m, 5H), 6.96 (s, 1H), 4.77 (s, 2H), 3.76 (s, 3H), 2.43 (s, 3H), 2.25 (s, 3H), 2.16 (s, 3H); ^13 ^C-NMR (CDCl_3_) *δ* 155.16, 150.60, 148.10, 143.55, 141.69, 136.96, 128.78 (2 C), 128.43, 128.05 (2 C), 126.58, 75.01, 52.71, 19.19, 14.98, 13.32.

#### 2.1.21. Methyl (5-hydroxy-3,4,6-trimethylpyridin-2-yl)carbamate (16a)

To a suspension of 10% palladium on activated carbon (12 mg) in a mixed solvent of CHCl_3_ (1.5 ml) and MeOH (1.5 ml) was added **15a** (55 mg, 0.18 mmol). The mixture was stirred with hydrogen balloon at room temperature for 1 h. After filtration of the reaction mixture through a Celite pad, the filtrate was concentrated. The residue was purified by silica gel column chromatography (CH_2_Cl_2_/MeOH = 20/1 to 5/1) to give **16a** (37 mg, 96%). White solid; TLC R_f_ 0.19 (CH_2_Cl_2_/MeOH = 15/1); m.p. 210 °C; MS (ESI) *m/z* 233 [M + Na]^+^; ^1^H-NMR (DMSO-*d_6_*) *δ* 9.02 (s, 1H), 8.53 (s, 1H), 3.57 (s, 3H), 2.27 (s, 3H), 2.11 (s, 3H), 2.00 (s, 3H); ^13 ^C-NMR (DMSO-*d_6_*) *δ* 155.20, 147.63, 141.39, 140.32, 133.75, 126.18, 51.47, 19.15, 14.14, 12.51.

#### 2.1.22. Methyl (5-hydroxy-3,4,6-trimethylpyridin-2-yl)carbamate (15 b)

To a solution of 5-benzyloxy-3,4,6-trimethylpyridin-6-amine (**5**, 70 mg, 0.29 mmol) in acetone (1 ml) was added K_2_CO_3_ (200 mg, 1.45 mmol) followed by dropwise addition of butyl chloroformate (184 µL, 1.45 mmol) at 0 °C. The mixture was stirred at room temperature for 24 h. The reaction mixture was concentrated then diluted with CH_2_Cl_2_ and washed with sat. NaHCO_3_ and brine. The CH_2_Cl_2_ layer was dried over MgSO_4_, filtered and concentrated. The residue was purified by silica gel column chromatography (CH_2_Cl_2_/MeOH = 30/1 to 20/1) to give **15 b** (90 mg, 91%). White solid; TLC R_f_ 0.33 (CH_2_Cl_2_/MeOH = 15/1); m.p. 81 °C; MS (ESI) *m/z* 399 [M + H]^+^; ^1^H-NMR (CDCl_3_) *δ* 7.51–7.33 (m, 5H), 7.08 (s, 1H), 4.77 (s, 2H), 4.14 (t, *J* = 6.6 Hz, 2H), 2.43 (s, 3H), 2.24 (s, 3H), 2.16 (s, 3H), 1.65 (dt, *J* = 14.8, 6.9 Hz, 2H), 1.40 (dq, *J* = 14.2, 7.2 Hz, 2H), 0.94 (t, *J* = 7.3 Hz, 3H); ^13 ^C-NMR (CDCl_3_) *δ* 154.83, 150.52, 147.90, 143.80, 141.66, 137.01, 128.75 (2 C), 128.38, 128.03 (2 C), 126.53, 75.02, 65.43, 31.11, 19.18, 19.08, 15.01, 13.85, 13.28.

#### 2.1.23. Butyl (5-hydroxy-3,4,6-trimethylpyridin-2-yl)carbamate (16 b)

To a suspension of 10% palladium on activated carbon (16 mg) in MeOH (5 ml) was added **15 b** (79 mg, 0.23 mmol). The mixture was stirred with hydrogen balloon at room temperature for 1 h. After filtration of the reaction mixture through a Celite pad, the filtrate was concentrated. The residue was dissolved in MeOH and filtered through a membrane filter and concentrated to give **16 b** (54 mg, 93%). White solid; TLC R_f_ 0.24 (CH_2_Cl_2_/MeOH = 15/1); m.p. 166 °C; MS (ESI) *m/z* 253 [M + H]^+^; ^1^H-NMR (DMSO-*d_6_*) *δ* 8.95 (s, 1H), 3.98 (t, *J* = 6.5 Hz, 2H), 2.27 (s, 3H), 2.11 (s, 3H), 1.99 (s, 3H), 1.55 (dt, *J* = 14.5, 6.6 Hz, 2H), 1.34 (dq, *J* = 14.1, 7.1 Hz, 2H), 0.89 (t, *J* = 7.3 Hz, 3H); ^13 ^C-NMR (DMSO-*d_6_*) *δ* 154.94, 147.82, 141.42, 140.33, 133.76, 126.26, 63.68, 30.80, 19.32, 18.65, 14.30, 13.70, 12.65.

#### 2.1.24. Isobutyl (5-(benzyloxy)-3,4,6-trimethylpyridin-2-yl)carbamate (15c)

To a solution of 5-benzyloxy-3,4,6-trimethylpyridin-6-amine (**5**, 30 mg, 0.12 mmol) in acetone (1 ml) was added K_2_CO_3_ (86 mg, 0.62 mmol) followed by dropwise addition of isobutyl chloroformate (24 µL, 0.19 mmol) at 0 °C. The mixture was stirred at room temperature for 24 h. The reaction mixture was concentrated and then diluted with CH_2_Cl_2_. It was washed with sat. NaHCO_3_ and brine. The CH_2_Cl_2_ solution was dried over MgSO_4_, filtered and concentrated. The residue was purified by silica gel column chromatography (CH_2_Cl_2_/MeOH = 40/1) to give **15c** (40 mg, 87%). White solid; TLC R_f_ 0.29 (CH_2_Cl_2_/MeOH = 15/1); m.p. 70 °C; MS (ESI) *m/z* 343 [M + H]^+^; ^1^H-NMR (CDCl_3_) *δ* 7.52–7.34 (m, 5H), 7.28 (s, 1H), 4.77 (s, 2H), 3.93 (d, *J* = 6.6 Hz, 2H), 2.44 (s, 3H), 2.24 (s, 3H), 2.17 (s, 3H), 2.04–1.87 (m, 1H), 0.94 (d, *J* = 6.7 Hz, 6H); ^13 ^C-NMR (CDCl_3_) *δ* 154.89, 150.50, 147.89, 143.87, 141.61, 137.01, 128.73 (2 C), 128.37, 128.02 (2 C), 126.53, 75.00, 71.63, 29.80, 28.09, 19.11 (d, 2 C), 15.00, 13.27.

#### 2.1.25. Isobutyl (5-(benzyloxy)-3,4,6-trimethylpyridin-2-yl)carbamate (16c)

To a suspension of 10% palladium on activated carbon (18 mg) in MeOH (5 ml) was added **15c** (91 mg, 0.27 mmol). The mixture was stirred with hydrogen balloon at room temperature for 6 h. After filtration of the reaction mixture through a Celite pad, the filtrate was concentrated. The residue was purified by silica gel column chromatography (CH_2_Cl_2_/MeOH = 30/1 to 20/1) to give **16c** (34 mg, 51%). White solid; TLC R_f_ 0.35 (CH_2_Cl_2_/MeOH = 15/1); m.p. 166 °C; MS (ESI) *m/z* 253 [M + H]^+^; ^1^H-NMR (DMSO-*d_6_*) *δ* 8.89 (s, 1H), 8.44 (s, 1H), 3.78 (d, *J* = 6.6 Hz, 2H), 2.28 (s, 3H), 2.12 (s, 3H), 2.01 (s, 3H), 1.86 (dt, *J* = 13.3, 6.6 Hz, 1H), 0.89 (d, *J* = 6.7 Hz, 6H); ^13 ^C-NMR (DMSO-*d_6_*) *δ* 154.85, 147.54, 141.30, 140.38, 133.66, 126.12, 69.84, 27.59, 19.10, 18.80 (2 C), 14.11, 12.47.

#### 2.1.26. 2-Chloroethyl (5-(benzyloxy)-3,4,6-trimethylpyridin-2-yl)carbamate (15d)

To a solution of 5-benzyloxy-3,4,6-trimethylpyridin-6-amine (**5**, 70 mg, 0.29 mmol) in acetone (1 ml) was added K_2_CO_3_ (200 mg, 1.45 mmol) followed by dropwise addition of 2-chloroethyl chloroformate (149 µL, 1.45 mmol) at 0 °C. The mixture was stirred at room temperature for 9 h. The reaction mixture was concentrated. The residue was diluted with CH_2_Cl_2_ and washed with sat. NaHCO_3_ and brine. The CH_2_Cl_2_ layer was dried over MgSO_4_, filtered and concentrated. The residue was purified by silica gel column chromatography (CH_2_Cl_2_/MeOH = 50/1 to 30/1) to give **15d** (84 mg, 83%). White solid; TLC R_f_ 0.21 (CH_2_Cl_2_/MeOH = 30/1); m.p. 128 °C; MS (ESI) *m/z* 349 [M + H]^+^; ^1^H-NMR (CDCl_3_) *δ* 7.51–7.34 (m, 5H), 4.78 (s, 2H), 4.44–4.34 (m, 2H), 3.78–3.66 (m, 2H), 2.44 (s, 3H), 2.25 (s, 3H), 2.17 (s, 3H); ^13 ^C-NMR (CDCl_3_) *δ* 154.05, 150.70, 148.22, 143.22, 141.80, 136.90, 128.79 (2 C), 128.45, 128.05 (2 C), 126.62, 75.02, 65.05, 42.06, 19.14, 14.96, 13.33.

#### 2.1.27. 2-Chloroethyl (5-(benzyloxy)-3,4,6-trimethylpyridin-2-yl)carbamate (16d)

To a suspension of **15d** (75 mg, 0.22 mmol) in CH_2_Cl_2_ (2 ml), was added pentamethylbenzene (96 mg, 0.65 mmol) followed by dropwise addition of boron trichloride (1 M in CH_2_Cl_2_, 0.32 ml) at 0 °C. The reaction mixture was stirred at 0 °C for 30 min, The mixture was then quenched with a mixed solvent of CHCl_3_/MeOH (9/1, 1.5 ml) and stirred at room temperature for 30 min. After concentration of the reaction mixture, the residue was purified by silica gel column chromatography (CHCl_3_/MeOH = 30/1 to 10/1) to give **16d** (51 mg, 92%). White solid; TLC R_f_ 0.18 (CH_2_Cl_2_/MeOH = 30/1); m.p. 163 °C; MS (ESI) *m/z* 259 [M + H]^+^; ^1^H-NMR (DMSO-*d_6_*) *δ* 9.11 (s, 1H), 8.48 (s, 1H), 4.30–4.22 (m, 2H), 3.85–3.78 (m, 2H), 2.28 (s, 3H), 2.12 (s, 3H), 2.02 (s, 3H); ^13 ^C-NMR (DMSO-*d_6_*) *δ* 154.28, 147.70, 141.39, 140.01, 133.74, 126.29, 64.06, 42.99, 19.11, 14.08, 12.48.

#### 2.1.28. 2-Methoxyethyl (5-(benzyloxy)-3,4,6-trimethylpyridin-2-yl)carbamate (15e)

To a solution of 5-benzyloxy-3,4,6-trimethylpyridin-6-amine (**5**, 70 mg, 0.29 mmol) in acetone (2 ml) was added K_2_CO_3_ (200 mg, 1.45 mmol) followed by dropwise addition of 2-methoxyethyl chloroformate (168 µL, 1.45 mmol) at 0 °C. The mixture was stirred at room temperature for 17 h and then concentrated. The residue was diluted with CH_2_Cl_2_ and washed with sat. NaHCO_3_ and brine. The CH_2_Cl_2_ layer was dried over MgSO_4_, filtered and concentrated. The residue was purified by silica gel column chromatography (CH_2_Cl_2_/MeOH = 40/1) to give **15e** (72 mg, 72%). White solid; TLC R_f_ 0.22 (CH_2_Cl_2_/MeOH = 30/1); m.p. 98 °C; MS (ESI) *m/z* 345 [M + H]^+^; ^1^H-NMR (CDCl_3_) *δ* 7.50–7.35 (m, 5H), 6.94 (s, 1H), 4.77 (s, 2H), 4.35–4.27 (m, 2H), 3.67–3.58 (m, 2H), 3.41 (s, 3H), 2.44 (s, 3H), 2.24 (s, 3H), 2.16 (s, 3H); ^13 ^C-NMR (CDCl_3_) *δ* 154.41, 150.65, 148.01, 143.46, 141.81, 136.98, 128.78 (2 C), 128.43, 128.06 (2 C), 126.51, 75.06, 70.90, 64.57, 59.11, 19.15, 14.91, 13.32.

#### 2.1.29. 2-Methoxyethyl (5-hydroxy-3,4,6-trimethylpyridin-2-yl)carbamate (16e)

To a suspension of 10% palladium on activated carbon (19 mg) in MeOH (5 ml) was added **15e** (95 mg, 0.28 mmol). The mixture was stirred with hydrogen balloon at room temperature for 1 h. After filtration of the reaction mixture through a Celite pad, the filtrate was concentrated. The residue was dissolved in MeOH and filtered through a membrane filter. The filtrate was concentrated to give **16e** (70 mg, 99%). White solid; TLC R_f_ 0.16 (CH_2_Cl_2_/MeOH = 30/1); m.p. 147 °C; MS (ESI) *m/z* 255 [M + H]^+^; ^1^H-NMR (DMSO-*d_6_*) *δ* 9.01 (s, 1H), 8.52 (br s, 1H), 4.11 (dd, *J* = 5.4, 3.9 Hz, 2H), 3.52 (dd, *J* = 5.4, 4.0 Hz, 2H), 3.27 (s, 3H), 2.28 (s, 3H), 2.12 (s, 3H), 2.00 (s, 3H); ^13 ^C-NMR (DMSO-*d_6_*) *δ* 154.74, 147.70, 141.40, 140.31, 133.77, 126.35, 70.29, 63.20, 58.05, 19.31, 14.25, 12.63.

#### 2.1.30. N-(5-(benzyloxy)-3,4,6-trimethylpyridin-2-yl)methanesulfonamide (17a)

To a solution of 5-benzyloxy-3,4,6-trimethylpyridin-6-amine (**5**, 50 mg, 0.21 mmol) in pyridine (2 ml) was added methanesulfonyl chloride (48 µL, 0.62 mmol). The mixture was stirred at room temperature for 24 h and then concentrated. The residue was diluted with CH_2_Cl_2_ and washed with brine. The CH_2_Cl_2_ layer was dried over MgSO_4_, filtered and concentrated. The residue was purified by silica gel column chromatography (CH_2_Cl_2_/MeOH = 40/1 to 20/1) to give **17a** (34 mg, 52%). White solid; TLC R_f_ 0.35 (Hexanes/EtOAc = 1/1); m.p. 122 °C; MS (ESI) *m/z* 321 [M + H]^+^; ^1^H-NMR (CDCl_3_) *δ* 9.23 (br, s, 1H), 7.52–7.29 (m, 5H), 4.75 (s, 2H), 3.23 (s, 3H), 2.33 (s, 3H), 2.23 (s, 3H), 2.15 (s, 3H); ^13 ^C-NMR (CDCl_3_) *δ* 147.81, 145.88, 144.54, 140.41, 136.48, 128.86 (2 C), 128.66, 128.23 (2 C), 123.99, 75.68, 42.92, 17.18, 13.77, 13.74.

#### 2.1.31. N-(5-Hydroxy-3,4,6-trimethylpyridin-2-yl)methanesulfonamide (18a)

To a suspension of 10% palladium on activated carbon (5 mg) in a mixed solvent of MeOH (2.5 ml) and CH_2_Cl_2_ (2.5 ml) was added **17a** (26 mg, 0.08 mmol). The mixture was stirred with hydrogen balloon at room temperature for 1 h. After filtration of the reaction mixture through a Celite pad, the filtrate was concentrated. The residue was dissolved in MeOH and filtered through a membrane filter and concentrated to give **18a** (19 mg, 100%). Pale yellow solid; TLC R_f_ 0.2 (CH_2_Cl_2_/MeOH = 20/1); m.p. 142 °C; MS (ESI) *m/z* 231 [M + H]^+^; ^1^H-NMR (DMSO-*d_6_*) *δ* 9.00 (s, 2H), 3.20 (s, 3H), 2.30 (s, 3H), 2.11 (s, 3H), 2.10 (s, 3H); ^13 ^C-NMR (DMSO-*d_6_*) *δ* 147.22, 141.11, 140.84, 134.54, 124.95, 42.20, 19.48, 14.07, 12.69.

#### 2.1.32. N-(5-(benzyloxy)-3,4,6-trimethylpyridin-2-yl)-4-methylbenzenesulfonamide (17 b)

To a solution of 5-benzyloxy-3,4,6-trimethylpyridin-6-amine (**5**, 100 mg, 0.41 mmol) in pyridine (2 ml) was added *p*-toluenesulfonyl chloride (87 mg, 0.45 mmol). The mixture was stirred at room temperature for 2 h and then concentrated. The residue was diluted with CH_2_Cl_2_ and washed with brine. The CH_2_Cl_2_ layer was dried over MgSO_4_, filtered and concentrated. The residue was purified by silica gel column chromatography (Hexanes/EtOAc = 3/1) to give **17 b** (82 mg, 50%). White solid; TLC R_f_ 0.34 (Hexanes/EtOAc = 2/1); m.p. 198 °C; MS (ESI) *m/z* 397 [M + H]^+^; ^1^H-NMR (CDCl_3_) *δ* 7.83 (d, *J* = 8.3 Hz, 2H), 7.43–7.33 (m, 5H), 7.24 (d, *J* = 8.0 Hz, 2H), 4.72 (s, 2H), 2.39 (s, 3H), 2.25 (s, 3H), 2.21 (s, 3H), 2.16 (s, 3H); ^13 ^C-NMR (CDCl_3_) *δ* 149.33, 146.34, 144.21, 142.36, 140.62, 136.70, 136.15, 129.26 (2 C), 128.88 (2 C), 128.76, 128.29 (2 C), 126.54 (2 C), 125.87, 75.93, 21.62, 15.88, 14.06, 13.85.

#### 2.1.33. N-(5-Hydroxy-3,4,6-trimethylpyridin-2-yl)-4-methylbenzenesulfonamide (18 b)

To a suspension of 10% palladium on activated carbon (26 mg) in MeOH (10 ml) was added **17 b** (130 mg, 0.33 mmol). The mixture was stirred with hydrogen balloon at room temperature for 2 h. After filtration of the reaction mixture through a Celite pad, the filtrate was concentrated. The residue was dissolved in MeOH and filtered through a membrane filter and concentrated to give **18 b** (86 mg, 86%). White solid; TLC R_f_ 0.21 (Hexanes/EtOAc = 1/1); m.p. 135 °C; MS (ESI) *m/z* 307 [M + H]^+^; ^1^H-NMR (DMSO-*d_6_*) *δ* 9.13 (br, s, 2H), 7.72 (d, *J* = 8.3 Hz, 2H), 7.32 (d, *J* = 8.2 Hz, 2H), 2.36 (s, 3H), 2.12 (s, 3H), 2.10 (s, 3H), 2.09 (s, 3H); ^13 ^C-NMR (DMSO-*d_6_*) *δ* 146.78, 141.98, 140.59, 140.41, 139.53, 134.66, 128.71 (2 C), 127.32 (2 C), 124.58, 20.95, 18.72, 14.05, 12.61.

#### 2.1.34. N-(5-(benzyloxy)-3,4,6-trimethylpyridin-2-yl)naphthalene-1-sulphonamide (17c)

To a solution of 5-benzyloxy-3,4,6-trimethylpyridin-6-amine (**5**, 50 mg, 0.21 mmol) in CH_2_Cl_2_ (1 ml) was added 1-napthalenesulfonyl chloride (70 mg, 0.31 mmol) followed by addition of triethylamine (57 µL, 0.41 mmol). The mixture was stirred at room temperature for 70 h. The reaction mixture was diluted with CH_2_Cl_2_ and washed with sat. NaHCO_3_ and brine. The CH_2_Cl_2_ layer was dried over MgSO_4_, filtered and concentrated. The residue was purified by silica gel column chromatography (Hexanes/EtOAc = 15/1 to 2/1) to give **17c** (54 mg, 61%). Pale yellow solid; TLC R_f_ 0.31 (Hexanes/EtOAc = 2/1);) ; m.p. 153 °C; MS (ESI) *m/z* 433 [M + H]^+^; ^1^H-NMR (CDCl_3_) *δ* 11.85 (br s, 1H), 9.03 (d, *J* = 8.5 Hz, 1H), 8.26 (dd, *J* = 7.3, 1.0 Hz, 1H), 7.96 (d, *J* = 8.2 Hz, 1H), 7.88 (d, *J* = 7.9 Hz, 1H), 7.65 (ddd, *J* = 8.5, 6.9, 1.4 Hz, 1H), 7.56 (dd, *J* = 11.0, 4.0 Hz, 1H), 7.50–7.43 (m, 1H), 7.43–7.29 (m, 5H), 4.69 (s, 2H), 2.23 (s, 3H), 2.18 (s, 3H), 2.12 (s, 3H); ^13 ^C-NMR (CDCl_3_) *δ* 160.04, 150.54, 147.41, 143.15, 139.48, 136.05, 134.45, 132.93, 128.90 (2 C), 128.82, 128.75, 128.47, 128.31 (2 C), 127.54, 126.78, 126.70, 126.63, 126.43, 124.06, 76.11, 15.30, 14.19, 13.68.

#### 2.1.35. N-(5-Hydroxy-3,4,6-trimethylpyridin-2-yl)naphthalene-1-sulphonamide (18c)

To a suspension of 10% palladium on activated carbon (22 mg) in MeOH (5 ml) was added **17c** (109 mg, 0.25 mmol). The mixture was stirred with hydrogen balloon at room temperature for 8 h. After filtration of the reaction mixture through a Celite pad, the filtrate was concentrated. The residue was purified by silica gel column chromatography (Hexanes/EtOAc = 2/1 to 1/1) to give **18c** (77 mg, 89%). Pale yellow solid; TLC R_f_ 0.32 (CH_2_Cl_2_/MeOH = 15/1); m.p. 95 °C; MS (ESI) *m/z* 343 [M + H]^+^; ^1^H-NMR (CDCl_3_) *δ* 8.92 (d, *J* = 8.5 Hz, 1H), 8.17 (dd, *J* = 7.3, 0.9 Hz, 1H), 7.90 (d, *J* = 8.2 Hz, 1H), 7.83 (d, *J* = 7.9 Hz, 1H), 7.68–7.56 (m, 1H), 7.52 (dd, *J* = 11.0, 4.0 Hz, 1H), 7.47–7.35 (m, 1H), 2.27 (s, 3H), 2.14 (s, 3H), 2.04 (s, 3H); ^13 ^C-NMR (CDCl_3_) *δ* 148.37, 144.31, 141.04, 139.26, 134.40, 132.99, 129.12, 128.60, 128.56, 127.52, 126.63, 126.46, 126.42, 125.67, 124.04, 15.50, 13.93, 13.59.

#### 2.1.36. N-(5-(benzyloxy)-3,4,6-trimethylpyridin-2-yl)-4-(trifluoromethyl)benzenesulfonamide (17d)

To a solution of 5-benzyloxy-3,4,6-trimethylpyridin-6-amine (**5**, 150 mg, 0.62 mmol) in pyridine (3 ml) was added 4-(trifluoromethyl)benzenesulfonyl chloride (227 mg, 0.93 mmol). The mixture was stirred at room temperature for 24 h and then concentrated. The residue was diluted with CH_2_Cl_2_ and washed with sat. NaHCO_3_ and brine. The CH_2_Cl_2_ layer was dried over MgSO_4_, filtered and concentrated. The residue was purified by silica gel column chromatography (Hexanes/EtOAc = 3/1) to give **17d** (231 mg, 83%). White solid; TLC R_f_ 0.33 (Hexanes/EtOAc = 3/1); m.p. 131 °C; MS (ESI) *m/z* 451 [M + H]^+^; ^1^H-NMR (CDCl_3_) *δ* 11.72 (s, 1H), 8.07 (d, *J* = 8.2 Hz, 2H), 7.69 (d, *J* = 8.3 Hz, 2H), 7.45–7.32 (m, 5H), 4.74 (s, 2H), 2.27 (s, 3H), 2.24 (s, 3H), 2.16 (s, 3H); ^13 ^C-NMR (CDCl_3_) *δ* 150.14, 147.69, 143.80, 135.98, 135.23, 133.21 (q, *J* = 32.9 Hz), 128.90 (2 C), 128.84, 128.31 (2 C), 126.70 (4 C), 126.27, 125.81 (q, *J* = 3.7 Hz), 121.47, 76.10, 15.52, 14.23, 13.69.

#### 2.1.37. N-(5-Hydroxy-3,4,6-trimethylpyridin-2-yl)-4-(trifluoromethyl)benzenesulfonamide (18d)

To a suspension of 10% palladium on activated carbon (27 mg) in MeOH (5 ml) was added **17d** (146 mg, 0.32 mmol). The mixture was stirred with hydrogen balloon at room temperature for 4 h. After filtration of the reaction mixture through a Celite pad, the filtrate was concentrated. The residue was dissolved in MeOH and filtered through a membrane filter and concentrated to give **18d** (82 mg, 70%). White solid; TLC R_f_ 0.32 (CH_2_Cl_2_/MeOH = 15/1); m.p. 165 °C; MS (ESI) *m/z* 361 [M + H]^+^; ^1^H-NMR (DMSO-*d_6_*) *δ* 10.17 (s, 1H), 8.55 (s, 1H), 8.03 (d, *J* = 8.3 Hz, 2H), 7.93 (d, *J* = 8.4 Hz, 2H), 2.12 (s, 3H), 2.10 (s, 3H), 2.07 (s, 3H); ^13 ^C-NMR (DMSO-d_6_) *δ* 146.89, 146.42, 140.31, 131.62 (dd, *J* = 64.2, 32.1 Hz), 128.17 (2 C), 125.83, 125.58 (q, *J* = 3.8 Hz), 124.84 (2 C), 121.50, 18.40, 13.93, 12.66.

#### 2.1.38. N-(5-(benzyloxy)-3,4,6-trimethylpyridin-2-yl)-4-nitrobenzenesulfonamide (17e)

To a solution of 5-benzyloxy-3,4,6-trimethylpyridin-6-amine (**5**, 100 mg, 0.41 mmol) in pyridine (2 ml) was added 4-nitrobenzenesulfonyl chloride (110 mg, 0.50 mmol). The mixture was stirred at room temperature for 4 h and then concentrated. The residue was diluted with CH_2_Cl_2_ and washed with sat. NaHCO_3_ and brine. The CH_2_Cl_2_ layer was dried over MgSO_4_, filtered and concentrated. The residue was purified by silica gel column chromatography (Hexanes/EtOAc = 4/1) to give **17e** (126 mg, 71%). Yellow solid; TLC R_f_ 0.25 (Hexanes/EtOAc = 2/1); m.p. 62 °C; MS (ESI) *m/z* 428 [M + H]^+^; ^1^H-NMR (CDCl_3_) *δ* 11.86 (br s, 1H), 8.25 (d, *J* = 8.7 Hz, 2H), 8.10 (d, *J* = 8.8 Hz, 2H), 7.49–7.31 (m, 5H), 4.74 (s, 2H), 2.28 (s, 3H), 2.24 (s, 3H), 2.14 (s, 3H); ^13 ^C-NMR (CDCl_3_) *δ* 150.09, 149.30, 148.24, 143.85, 135.85, 135.09, 135.07, 128.86 (2 C), 128.83, 128.27 (2 C), 127.28 (2 C), 126.36, 123.97 (2 C), 76.10, 15.44, 14.27, 13.63.

#### 2.1.39. N-(5-Hydroxy-3,4,6-trimethylpyridin-2-yl)-4-nitrobenzenesulfonamide (18e)

To a suspension of **17e** (74 mg, 0.17 mmol) in CH_2_Cl_2_ (2 ml), was added pentamethylbenzene (77 mg, 0.52 mmol) followed by dropwise addition of boron trichloride (1 M in CH_2_Cl_2_, 0.35 ml) at 0 °C. The reaction mixture was stirred at 0 °C for 15 min then quenched with a mixed solvent of CHCl_3_/MeOH (9/1, 1.5 ml) and stirred at room temperature for 30 min. After concentration of the reaction mixture, the residue was purified by silica gel column chromatography (CH_2_Cl_2_/MeOH = 20/1) to give **18e** (47 mg, 81%). Yellow solid; TLC R_f_ 0.29 (Hexanes/EtOAc = 2/1); m.p. 199 °C; MS (ESI) *m/z* 388 [M + H]^+^; ^1^H-NMR (CD_3_OD) *δ* 8.36 − 8.27 (m, 2H), 8.09 − 8.00 (m, 2H), 2.28 (s, 3H), 2.19 (s, 3H), 2.10 (s, 3H); ^13 ^C-NMR (CD_3_OD) *δ* 150.91, 150.53, 147.15, 144.33, 141.49, 137.52, 129.19 (2 C), 127.54, 124.85 (2 C), 16.80, 14.01, 13.35.

#### 2.1.40. 2-Bromo-5-methoxy-3,4,6-trimethylpyridine (19)

To a solution of 6-bromo-2,4,5-trimethylpyridin-3-ol (**3**, 216 mg, 1.0 mmol) of CH_3_CN (10 ml) were added K_2_CO_3_ (207 mg, 1.5 mmol) and iodomethane (0.12 ml, 2.0 mmol). The mixture was stirred at 50 °C for 16 h and then cooled to room temperature. It was diluted with EtOAc and washed with 1 M HCl, water and brine successively. The EtOAc solution was dried over MgSO_4_, filtered and concentrated. The residue was purified by silica gel column chromatography (Hexanes/EtOAc = 9/1) to give **19** (154 mg, 67%). Yellow oil; TLC R_f_ 0.37 (Hexanes/EtOAc = 9/1); MS (ESI) *m/z* 230 [M + H]^+^; ^1^H-NMR (CDCl_3_) *δ* 3.67 (s, 3H), 2.44 (s, 3H), 2.30 (s, 3H), 2.24 (s, 3H); ^13 ^C-NMR (CDCl_3_) *δ* 152.76, 150.07, 141.29, 137.88, 131.95, 60.33, 18.88, 18.77, 13.50.

#### 2.1.41. N-(diphenylmethylene)-5-methoxy-3,4,6-trimethylpyridin-2-amine (20)

To a suspension of **19** (155 mg, 0.67 mmol) in toluene (5 ml) were added BINAP (42 mg, 0.07 mmol), Pd_2_(dba)_3_ (31 mg, 0.03 mmol), NaOBu*^t^* (71 mg, 0.74 mmol), and benzophenone imine (0.11 ml, 0.67 mmol). The mixture was stirred at 115 °C for 4 h and then cooled to room temperature. It was diluted with EtOAc and washed with water and brine. The EtOAc layer was dried over MgSO_4_, filtered and concentrated. The residue was purified by silica gel column chromatography (Hexanes/EtOAc = 4/1) to give **20** (170 mg, 77%). Yellow solid; TLC R_f_ 0.15 (Hexanes/EtOAc = 4/1); m.p. 95 °C; MS (ESI) *m/z* 331 [M + H]^+^; ^1^H-NMR (CDCl_3_) *δ* 7.80 − 7.36 (m, 10H), 3.66 (s, 3H), 2.52 (s, 3H), 2.29 (s, 3H), 2.10 (s, 3H); ^13 ^C-NMR (CDCl_3_) *δ* 169.36, 156.96, 149.30, 146.80, 139.56, 139.23, 136.89, 130.80, 129.60, 128.89, 128.56, 127.98, 127.53, 119.56, 60.36, 18.74, 13.79, 12.37.

#### 2.1.42. 5-Methoxy-3,4,6-trimethylpyridin-2-amine (21)

A solution of **20** (168 mg, 0.51 mmol) in a mixed solvent of THF/MeOH (1/10, 5.5 ml) was cooled to 0 °C. A mixed solution of CH_3_COCl/MeOH (1/10, 1 ml) was slowly added to the mixture. The reaction mixture was stirred at room temperature for 18 h and then concentrated. The residue was diluted with EtOAc and water. The EtOAc layer separated was extracted with water. To the combined aqueous solution was added 1 M NaOH until the pH of the solution reached 10. After extraction of the mixture with EtOAc, the EtOAc solution was dried over MgSO_4_, filtered and concentrated. The residue was purified by silica gel column chromatography (CH_2_Cl_2_/MeOH = 30/1) to give **21** (54 mg, 64%). Grey solid; TLC R_f_ 0.15 (CH_2_Cl_2_/MeOH = 20/1); m.p. 84 °C; MS (ESI) *m/z* 167 [M + H]^+^; ^1^H-NMR (DMSO-*d_6_*) *δ* 5.22 (s, 2H), 3.52 (s, 3H), 2.17 (s, 3H), 2.06 (s, 3H), 1.91 (s, 3H); ^13 ^C-NMR (CDCl_3_) *δ* 152.25, 146.62, 145.79, 139.72, 113.51, 60.53, 18.45, 13.05, 12.42.

#### 2.1.43. 1–(3,4-Dichlorophenyl)-3–(5-methoxy-3,4,6-trimethylpyridin-2-yl)thiourea (22a)

To a solution of **21** (25 mg, 0.15 mmol) in EtOH (2 ml) was added 3,4-dichlorophenyl isothiocyanate (24 μL, 0.16 mmol). The reaction mixture was stirred at room temperature for 48 h and then concentrated. The residue was purified by recrystallization over EtOH to give **22a** (39 mg, 70%). White solid; TLC R_f_ 0.63 (Hexanes/EtOAc = 4/1); m.p. 176 °C; MS (ESI) *m/z* 370 [M + H]^+^; ^1^H-NMR (DMSO-*d_6_*) *δ* 13.05 (s, 1H), 9.54 (s, 1H), 8.22 (s, 1H), 7.81 (d, *J* = 8.0 Hz, 1H), 7.59 (t, *J* = 7.6 Hz, 1H), 7.55 − 7.47 (m, 1H), 3.66 (s, 3H), 2.41 (s, 3H), 2.21 (s, 3H), 2.19 (s, 3H); ^13 ^C-NMR (DMSO-*d_6_*) *δ* 178.41, 149.52, 145.53, 145.11, 141.92, 139.20, 130.49, 130.22, 126.54, 124.63, 123.60, 121.50, 60.21, 18.43, 13.23, 12.67.

#### 2.1.44. 1–(5-Methoxy-3,4,6-trimethylpyridin-2-yl)-3–(3-(trifluoromethyl)phenyl)thiourea (22 b)

To a solution of **21** (25 mg, 0.15 mmol) in EtOH (2 ml) was added 3-(trifluoromethyl)phenyl isothiocyanate (24 μL, 0.16 mmol). Reaction mixture was stirred at room temperature for 48 h and then concentrated. The residue was purified with silica flash column chromatography (Hexanes/EtOAc = 4/1) to give **22 b** (30 mg, 54%). White solid; TLC R_f_ 0.46 (Hexanes/EtOAc = 4/1); m.p. 140 °C; MS (ESI) *m/z* 370 [M + H]^+^; ^1^H-NMR (DMSO-*d*_6_) *δ* 13.05 (s, 1H), 9.54 (s, 1H), 8.22 (s, 1H), 7.81 (d, *J* = 8.0 Hz, 1H), 7.59 (t, *J* = 7.6 Hz, 1H), 7.55 − 7.47 (m, 1H), 3.66 (s, 3H), 2.41 (s, 3H), 2.21 (s, 3H), 2.19 (s, 3H); ^13 ^C-NMR (DMSO-*d*_6_) *δ* 178.52, 149.47, 145.59, 145.06, 141.90, 139.91, 129.67, 128.98 (q, *J* = 31.6 Hz), 127.27, 125.37, 122.67, 121.27, 119.61, 60.20, 18.42, 13.20, 12.66.

### 2.2. Materials for biology

RPMI-1640, foetal bovine serum (FBS), penicillin/streptomycin and trizol reagent were obtained from Invitrogen Life Technologies (Carlsbad, CA, USA). Trypsin/EDTA was purchased from Clonetics, Inc. (Walkersville, MD, USA). Recombinant human TNF-α was procured from R & D system Inc, (Minneapolis, MN, USA). BCECF/AM (acetoxymethyl ester) was received from Molecular probes (Eugene, Oregon, USA). Antibodies directed against TNF-α, IL-1β, Claudin-2, NLRP3, Claudin-3 and Caspase-1 were purchased from Abcam (Cambridge, MA, USA). Antibodies against IL-6 and IL-10 were obtained from Abbiotec (San Diego, CA, USA). E-cadherin, ZO-1, IL-18 and I-κB antibodies were purchased from Santa Cruz Biotechnology (Santa Cruz, CA, USA) while NF-κB, TGFβ and phospho-I-κB antibodies were purchased from Cell Signalling Technology Inc. (Beverly, MA, USA).

### 2.3. Cell culture

HT-29 human colonic epithelial cell line and U937 human pre-monocytic cell line were obtained from the American Type Culture Collection (Manassas, VA, USA). HT-29 cells and U937 cells were cultured in RPMI-1640 media containing 10% FBS, 100 IU/ml of penicillin and 100 μg/mL of streptomycin. Cells were maintained at 37 °C in 5% CO_2_ in humidifier incubator.

### 2.4. Measurements of monocyte adhesion to epithelial cell layer

Adhesion of monocytes to colon epithelial cell layer was measured by using U937 pre-monocytic cells prelabeled with 2′,7′-bis(2-carboxyethyl)-5(6)-carboxylfluorescein acetoxymethyl ester (BCECF/AM, 10 µg/ml) as previously reported^21^^,^^22^ with slight modification. HT-29 cells (2 × 10^5^ cells/well) cultured in 48-well plates were pre-treated with compounds for 1 h. The prelabeled U937 cells were added on the monolayer of HT-29 cells, and they were treated with TNF-α (10 ng/mL) for 3 h at 37 °C. Non adhering U937 cells were removed by washing thrice with PBS. Cells were lysed with 0.1% Triton X-100 in Tris (0.1 M), and fluorescence intensity was measured using Fluostar Optima microplate reader (BMG LABTECH GmbH, Germany) at an excitation and emission wavelengths of 485 and 520 nm, respectively.

### 2.5. TNBS-induced colitis in rats

Sprague-Dawley female rats (seven weeks old) were purchased from Orient Bio Inc (Gyeonggi, South Korea) and were acclimatised to the lab condition for 1 week by giving food and water provided *ad libitum*. Rats were fasted for 24 h before TNBS injection (0.8 ml of 5% TNBS in 50% ethanol) into the lumen of the colon (8 cm proximal to the anus through the rectum) through an polyethylene catheter fitted onto a 1 ml syringe. Right after TNBS injection, rats were kept in vertical position for 1 min before being returned to the cage. Next day, sulfasalazine (300 mg/kg) or compounds (1 mg/kg) in corn oil was administered for up to 5 days. The doses of the drug were selected based on the previous studies^18^^,^^19^. On the sixth day, all the rats were sacrificed and macroscopic observations were evaluated for ulceration and severity of colitis. The colon tissues from 5 to 7 cm proximal to rectum were excised. The colon tissues were stored at −80 °C to examine the protein expression of inflammatory mediators, junctional molecules, and transcription factors.

The study protocol of the animal experiment was reviewed and approved beforehand by the Institutional Animal Care and Use Committee of Yeungnam University and were performed following the institutional guidelines of the Institute of Laboratory Animal Resources (1996), and of Yeungnam University for the care and use of animals (2009).

### 2.6. Measurement of myeloperoxidase level

Colon myeloperoxidase (MPO) level was measured by using MPO Rat ELISA kit (HK105-01, Hycult biotechnology, Netherland). Colon tissues were weighed, washed in cold 1× PBS (pH: 7.4), and homogenised in 500 μL of ice-cold lysis buffer (pH 7.4, 200 mM NaCl, 5 mM EDTA, 10 mM Tris, 10% glycerol, 1 mM PMSF, 1 µg/mL leupeptide, 28 µg/mL aprotinin) by using a Bead Blaster (Benchmark scientific, Edison, NJ, USA). MPO amount in the supernatant was measured by detecting the absorbance at 450 nm in microplate reader (Spectrostar Nano, BMG LABTECH, Ortenberg, Germany).

### 2.7. Protein extraction and Western blotting

Total protein from the colon was extracted by using radioimmunoprecipitation assay (RIPA) buffer (150 mM Sodium chloride, 1% Triton X-100, 0.5% Sodium deoxycholate, 0.1% SDS and 50 mM Tris adjusted to pH 8.0) containing 1X proteases and phosphatase inhibitor (Thermo Scientific, Rockford, USA). Cytosolic and nuclear proteins were extracted by using manufacturer’s instruction given by NE-PER nuclear and cytoplasmic extraction reagent kit (#78833, Thermo Scientific, Rockford, USA). Protein concentrations were measured using a BCA protein assay kit (Pierce, Rockford, IL, USA). Equal amounts of total proteins were then separated by SDS-PAGE and transferred onto Hybond ECL nitrocellulose membranes (Amersham Life Science, Buckinghamshire, UK) at 200 mA for 1 h. Blocking was done using 5% BSA in Tris-buffered saline (TBS)-Tween 20 (TBS-T) at 20 °C for 1 h. The membranes were incubated for 16 h at 4 °C with primary antibody diluted in TBS containing 3% BSA. After incubation, the membranes were washed three times with TBS-T. Next, the membrane was incubated for 1 h at 20 °C with horseradish peroxidase-conjugated secondary antibody in TBS. Immunoreactive proteins were visualised using an enhanced chemiluminescence (ECL) kit (Pierce, Rockford, IL, USA) and digitally processed using an LAS-4000 mini (Fuji, Japan). The membranes were stripped and re-probed with an actin antibody as a loading control.

### 2.8. Statistical analysis

Data are presented as the mean ± SEM, and were analysed by one way ANOVA followed by the Newman–Keuls comparison method using GraphPad Prism software (version 5.0, San Diego, CA, USA). P values less than 0.05 were considered statistically significant.

## 3. Results

### 3.1. Design and synthesis

**Scheme 1.**
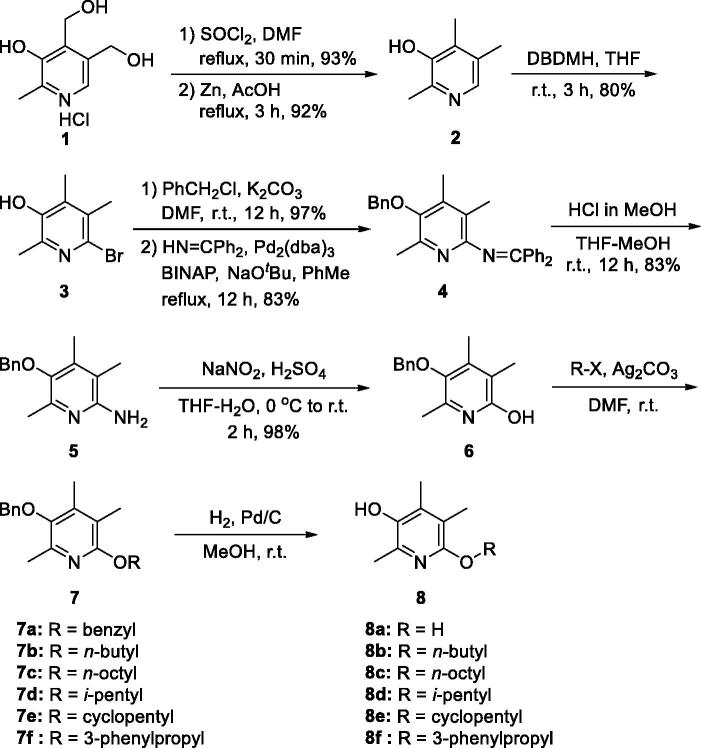
Synthesis of alkoxy-analogues **8**.

At first, we were curious about the effect of heteroatom at C(6)-position on activity and replaced nitrogen with oxygen to give 6-alkoxy analogues **8**. Synthesis of **8** was conducted *via* intermediate **5** which has been reported by us (Scheme 1)^15^. Briefly, two primary hydroxy groups of pyridoxine·HCl (**1**) was converted to chlorides using SOCl_2_ and catalytic amount of DMF. Then, two benzyl chloride groups were reductively removed with Zn and acetic acid under refluxing conditions to give 2,4,5-trimethylpyridin-3-ol (**2**). The C(6)-position was brominated using 1,3-dibromo-5,5-dimethylhydantoin (DBDMH) and the phenolic OH group of **3** was protected as benzyloxy group. Bromide on C(6)-position was replaced with benzophenone imine under Buchwald–Hartwig amination conditions to afford **4**. The imine group was then methanolyzed to give the free amino compound (**5**). The amino group of **5** was converted to hydroxy group via diazonium ion to give **6**, which was then treated with various alkyl halides to give alkoxy compounds **7**. Lastly, the benzyl protective group was removed by catalytic hydrogenolysis to give 6-alkoxy-2,4,5-trimethylpyridin-3-ol analogues **8**. The dihydroxy compound **8a** was obtained from the dibenzyloxy compound **7a**. A direct approach to obtain **8a** from **6** under the standard catalytic hydrogenolysis reaction conditions gave poor yield.

**Figure 2.**
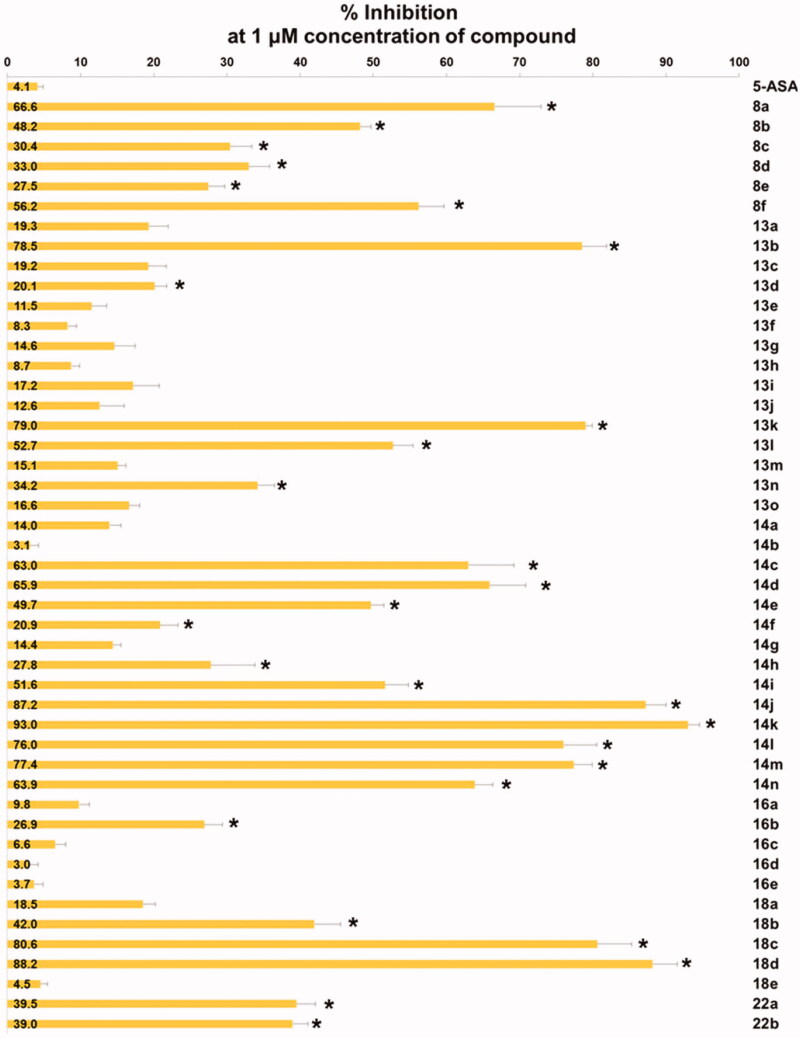
Inhibitory activity of the compounds against TNF-α-induced monocyte adhesion to colon epithelial cells. Data are shown as mean ± SEM of at least three independent experiments. *p < 0.05 compared to TNF-α-induced HT-29 cells.

Unfortunately, all alkoxy analogues (**8a**–**8f**) showed modest activity (29–67%) in our cell adhesion assay (Figure 2). Compound **8a** (67% inhibition) and **8f** (56% inhibition) showed 2.9- and 2.1-fold higher activity than the corresponding nitrogen counterparts whose inhibition level was 23% and 27%, respectively. Contrarily, compound **8e** (29% inhibition) showed 2.5-fold less activity than the corresponding nitrogen counterpart (73% inhibition)^18^. The rest of alkoxy analogues with alkyl groups (**8 b**, **8c**, **8d**) did not show distinct activity from 6-alkylamino analogues. It seems that nitrogen atom does not necessarily be replaced and might be better to remain on C(6)-position for the better topological polar surface area (tPSA) and calculated partition coefficient (cLogP). By locating nitrogen back to C(6)-position, about 10% of both tPSA and cLogP values can be restored compared to the alkoxy analogues. Therefore, we decided to explore structural diversity with nitrogen atom on the position. 
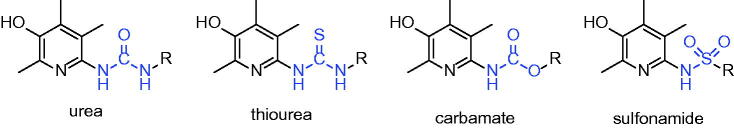


Now that we have reported that 6-(substituted)amino-2,4,5-trimethylpyridin-3-ols have high activity against colitis *in vitro* and *in vivo*, structural exploration with nitrogen on C(6)-position was designed to install ureido-, thioureido-, carbamato- and sulfonamido-linkage between pyridine ring and R group.

Described in Scheme 2 is the general scheme for the preparation of urea and thiourea analogues, **13** and **14**. Isocyanates (R − N = C = O)/isothiocyanates (R − N = C = S) were employed for the construction of urea/thiourea moieties. Two key intermediates, **5** and **10**, were used for the coupling reactions and the TBDPS-protected intermediate (**10**) was prepared by the synthetic procedure which we reported before^20^. In short, compound **10** was prepared from 2,4,5-trimethylpyridin-3-ol (**2**) by the following sequence; bromination of C(6)-position, protection of − OH with TBDPS group, palladium-catalyzed amination with benzophenone imine, and acidic methanolysis of the imine group. The coupled products with isocyanates and isothiocyanates, **11** and **12**, were then transformed the final compounds, **13** and **14**, by deprotection of *O*-benzyl or *O*-silyl group. For **14a** compound with − NH_2_ group, the precursor, *O*-benzyl protected compound **12a**, was obtained from *N*-benzoyl thiourea compound **12n** by a debenzoylation under basic hydrolysis conditions.

Scheme 2.Synthesis of ureido- and thioureido-analogues, **13** and **14**.

**Figure:**
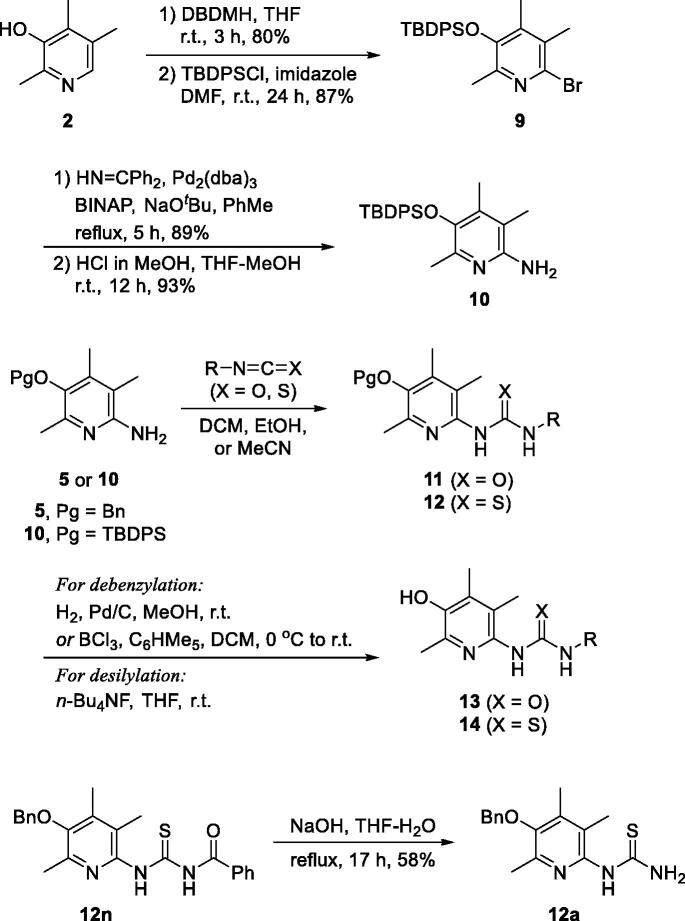

**Figure:**
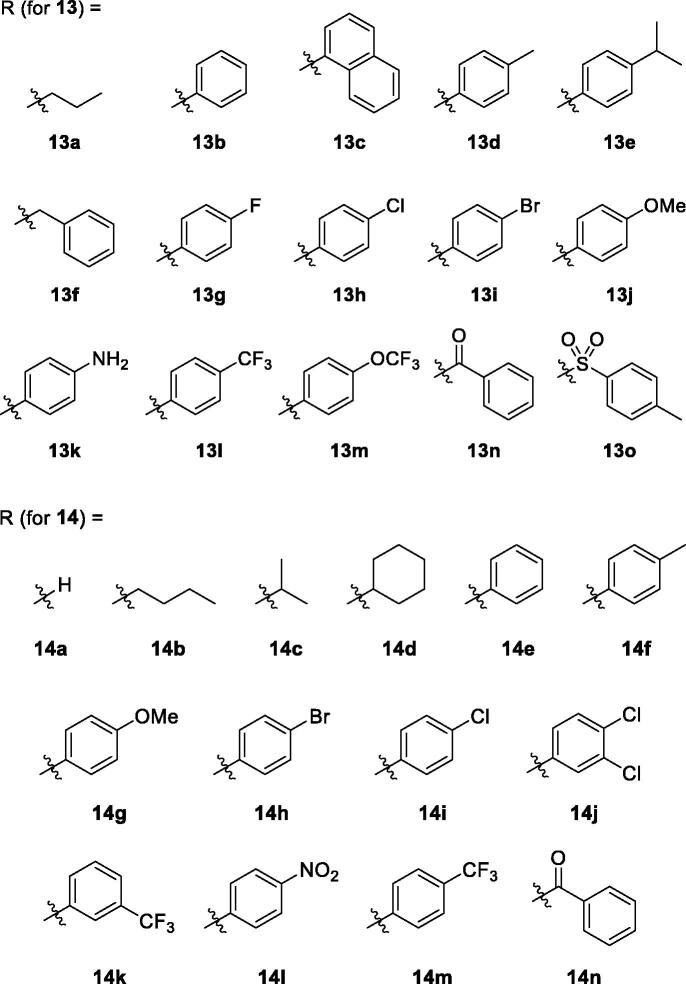

**Scheme 3.**
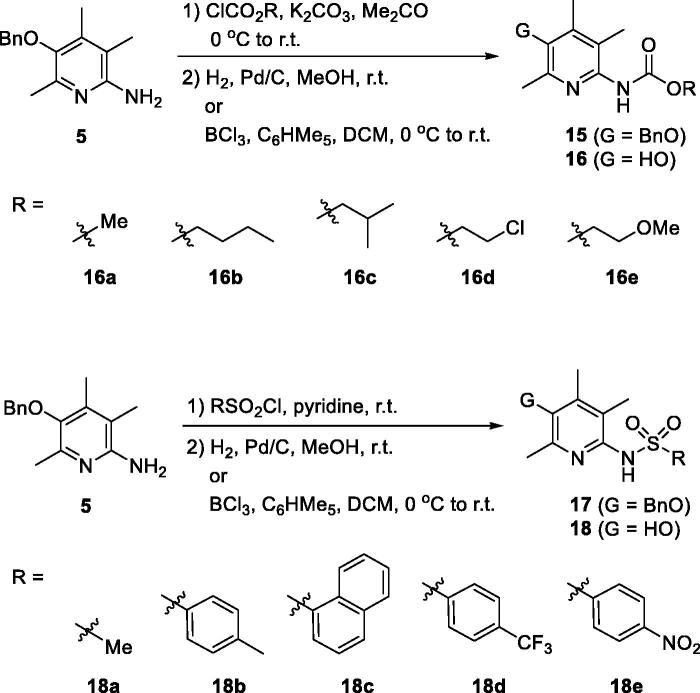
Synthesis of carbamato- and sulfonamido- analogues, **16** and **18**.

For the synthesis of sulfonamido- (**16**) and carbamato-analogues (**18**), the key intermediate **5** was treated with either chloroformates or sulphonyl chlorides to give **15** and **17**. They were then debenzylated to afford **16** and **18**, respectively (Scheme 3).

### 3.2. Inhibitory effects of 2,4,5-trimethylpyridin-3-ol derivatives on TNF-α-induced adhesion of monocytes to colonic epithelial cells

*In vitro* activity data of the alkoxy- (**8**), ureido- (**13**), thioureido- (**14**), carbamato- (**16**) and sulfonamido-analogues (**18**) are listed in Figure 2. As stated above, alkoxy analogues (**8**) showed activity in a somewhat similar range to the nitrogen counterparts. The best activity (67%) was seen in compound **8a** which has no substituent on C(6)-oxygen. Activity of urea (**13**) and thiourea (**14**) analogues are also in a similar range. Among the compounds with the same R group, the phenyl group is more active in urea analogue (**13 b**: 76.3%) than in thiourea analogue (**14e**: 49.7%). Both *p*-tolyl and *p*-methoxyphenyl groups showed similar and low activity both in urea analogues (**13d**: 20.1%, **13j**: 12.6%, respectively) and thiourea analogues (**14f**: 19.2%, **14 g**: 13.3%, respectively). IC_50_ values of some compounds of high % inhibition were measured to be 0.75 μM (**8f**), 0.41 μM (**13 b**), 0.40 μM (**14n**), 0.32 μM (**18d**), and 0.23 μM (**18c**). The order of these values is quite lined with the order of % inhibition measured at the fixed single concentration (1 μM).

**Scheme 4.**
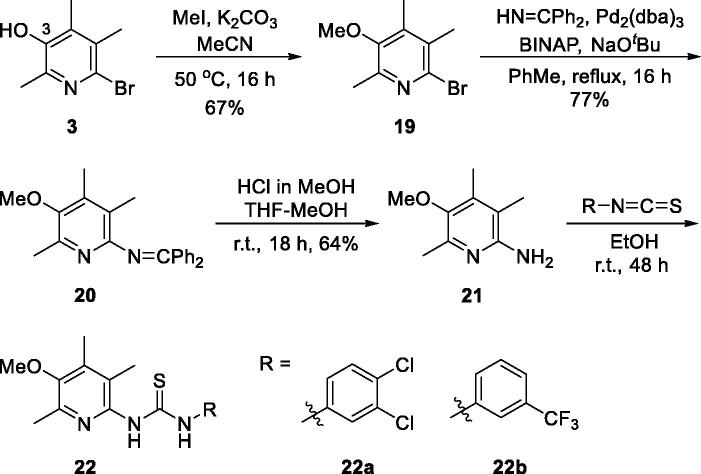
Synthesis of 3-methoxy analogues, **22**.

Next, we tried to investigate the role –OH group on C(3)-position on the activity. The hydroxy group is protected as methoxy group as shown in structure **22** to study if the OH group is essential (Scheme 4). Compounds **22a** and **22 b** are 3-methoxy analogues of **14j** and **14k**, respectively, which showed excellent activity in our *in vitro* assay as shown in Figure 1. The intermediate **3** is methylated with iodomethane and K_2_CO_3_ to give 3-methoxy compound **19**. Then, benzophenone imine was coupled under Buchwald-Hartwig amination conditions to afford imino compound **20** which in turn was hydrolysed to free amino compound **21**. The amino group of **21** was coupled with isothiocyanates to give **22a** and **22 b**. As shown in Figure 2, unfortunately, 3-methoxy analogues **22a** and **22 b** showed more than 2-fold less activity than corresponding 3-hydroxy analogues, **14j** and **14k**, respectively. It might imply that free hydroxyl group at 3-position is essential to the activity by contributing some sort of hydrogen bonding to a binding partner which is responsible for the activity. Further detail of the role of hydroxyl group will be investigated in subsequent studies.

### 3.3. Anti-inflammatory effects of seven 2,4,5-trimethylpyridin-3-ol derivatives on TNBS-induced rat colitis

**Figure 3.**
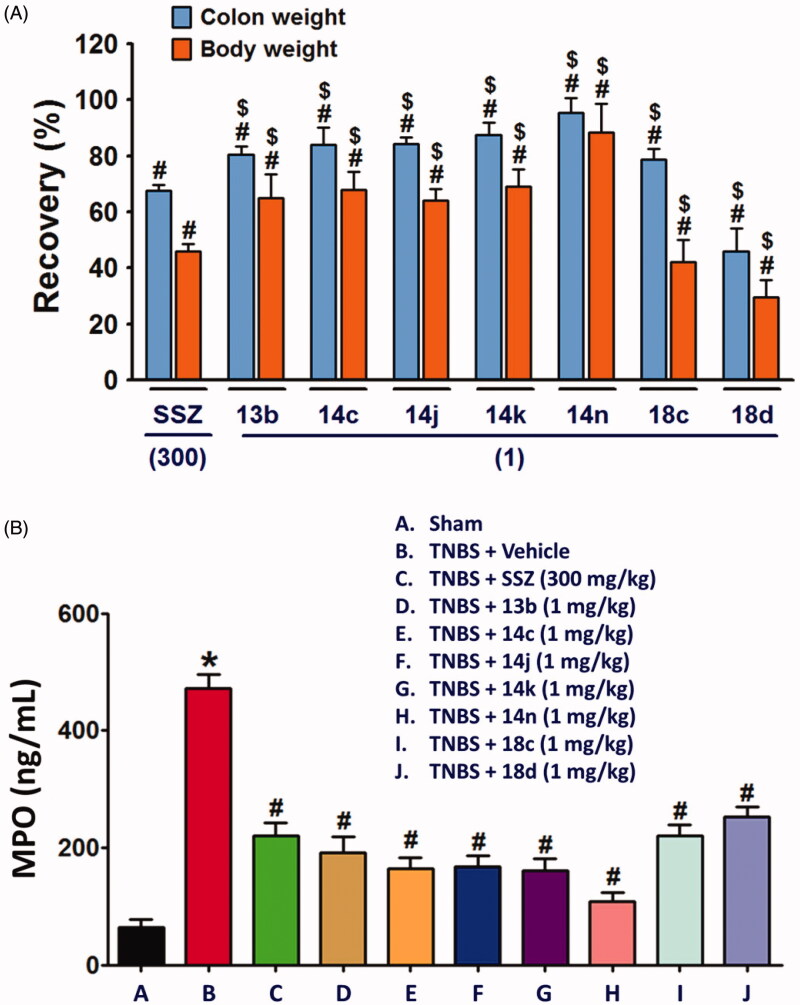
Recovery effects of compounds on TNBS-induced rat colitis, body and colon weights, and MPO activity. Colitis was induced by rectal administration of TNBS, and sham group received vehicle (50% ethanol) in the same route. Compounds (1 mg/kg) and SSZ (300 mg/kg) were given orally 1 day after TNBS treatment. Data represent the mean ± SEM for five rats per group. (A) Body weight was recorded daily from day 0 to day 5, and body weight recovery was calculated based on the last day measurement. Colon wet weight (distal 5–6 cm segment) was measured right after dissection of the colon. **^#^***p* < 0.05 compared to TNBS-treated group. **^$^***p* < 0.05 compared to SSZ-treated group. (B) MPO level of colon tissues *p < 0.05 compared to sham-operated control group. ^#^*p* < 0.05 compared to TNBS-treated group.

Next, we evaluated in vivo anti-inflammatory activity using TNBS-induced rat colitis model. Seven pyridin-3-ol compounds, **13 b**, **14c**, **14j**, **14k**, **14n**, **18c**, and **18d**, were selected for the *in vivo* efficacy evaluation along with sulfasalazine (SSZ) as the positive control drug. Colitis was induced by rectal administration of TNBS. Our seven pyridin-3-ol compounds along with SSZ were orally administered once a day for five days starting one-day after administration of TNBS at a dose of 1 mg/kg (for seven pyridin-3-ols) and 300 mg/kg (for SSZ), respectively. After treatment with TNBS, the rats showed signs of colitis, such as body weight decrease, bloody diarrhoea, and sluggish and weak movement. Compared to the sham-operated control group, the body weight of the TNBS-treated rats was significantly reduced and then increased gradually. Colon tissues in the TNBS-treated lesion site showed significant inflammation, as revealed by oedema and adhesion in gross morphology examination and increased wet weight per colon length. As a macroscopic marker to represent a disease phenotype, we measured the recovery level in body- and colon-weights upon drug treatment (Figure 3). Oral administration of rats with compounds, **13 b**, **14c**, **14j**, **14k**, **14n**, **18c**, or **18d** (1 mg/kg) significantly restored TNBS-induced body weight loss (Figure 3(A)). The recovery rate by the compounds (1 mg/kg) ranged from 25% to 98.9%, among which compound **14n** was the most excellent. The recovery rate of SSZ (300 mg/kg), a positive control, was 48.8%. Colon weight recovery was between 50.3% and 94.9%, which was better than the body weight recovery in most cases of oral administration of the compounds including sulfasalazine (Figure 3(A)). Compound **14n** (1 mg/kg) was the most effective in colon weight recovery as in the case of body weight recovery. In addition, the myeloperoxidase (MPO) level in colon tissues, which is directly related to neutrophil infiltration into tissues and serves as a biochemical marker of inflammation^23^, was examined. TNBS treatment induced a tremendous increase in MPO activity (Figure 4), and the increase was significantly suppressed by oral administration with compounds (1 mg/kg). The order of the compounds having excellent effects was **14n**, **14k**, **14c**, **14j**, **13 b, 18c**, and **18d**, demonstrating the significant efficacy of the new scaffold.

Figure 4.Inhibitory effects of compounds on expressions of inflammatory cytokines and epithelial junction molecules. Total protein extracts from colon tissues homogenates were used for detecting the protein expression of inflammatory mediators, TNFα, IL-6, and IL-1β, and junction molecules, E-cadherin, claudin-2, claudin-3, and ZO-1. The bar graphs represent the mean ± SEM of protein expression quantified in three independent experiments. *p < 0.05, compared with vehicle-treated control group. ^#^*p* < 0.05, compared with the TNBS-treated group.

**Figure:**
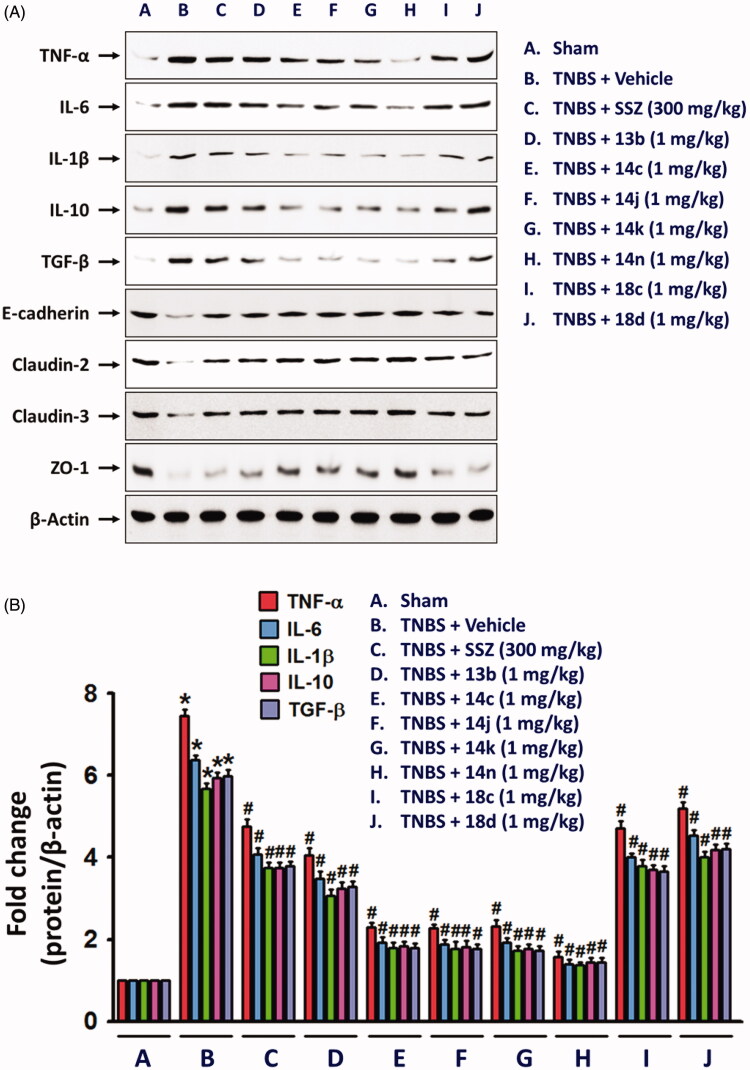

**Figure:**
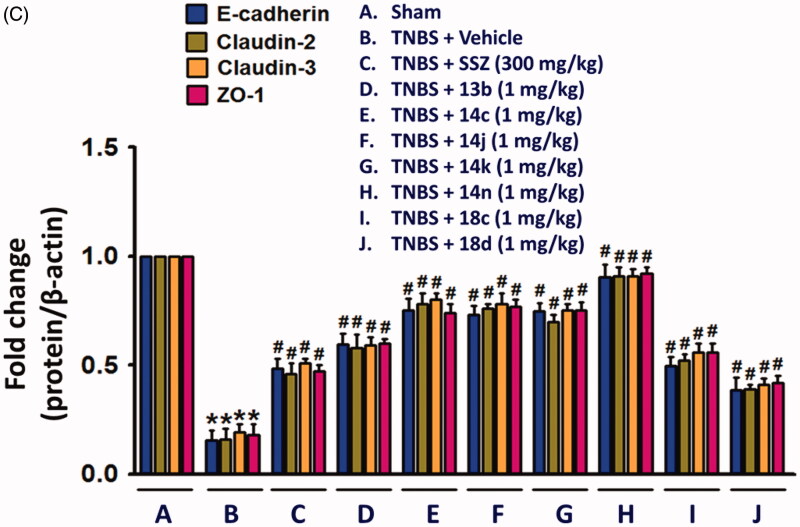

### 3.4. Suppressive effects of seven 2,4,5-trimethylpyridin-3-ol derivatives on TNBS-induced expression changes of inflammatory cytokines, junction molecules, inflammasome, and nuclear transcription factor-κB

Although the fundamental molecular targets of the compounds responsible for the phenotype are yet to be investigated, we further examined in this study the inhibitory effects of the compounds on the expression levels of cytokines responsible for the inflammatory response. It will provide more consistency to our mechanism based on the assay against TNF-α-induced cell adhesion. In rat colon tissues, TNBS treatment significantly increased the expression of TNF-α, IL-6 and IL-1β. The expression level of IL-10 and TGF-β which are known to be secreted by M2 macrophages for termination of inflammation and tissue repair^24^^,^^25^, were also significantly increased in the inflamed colon tissues (Figure 4(A,B)). In contrast, level of colon epithelial junction molecules, E-cadherin, Claudin-2, Claudin-3 and ZO-1 was significantly reduced by TNBS (Figure 4(A,C)). However, oral administration of compounds, **13b**, **14c**, **14j**, **14k**, **14n**, **18c**, or **18d** (1 mg/kg) significantly restored the TNBS-altered protein expressions of junctional molecules. All these changes consistently and strongly support the anti-IBD activity of our compounds. Indeed, the inhibitory effects of compounds, **14c**, **14j**, **14k**, and **14n** (1 mg/kg) was much stronger than that of 300 mg/kg SSZ.

**Figure 5.**
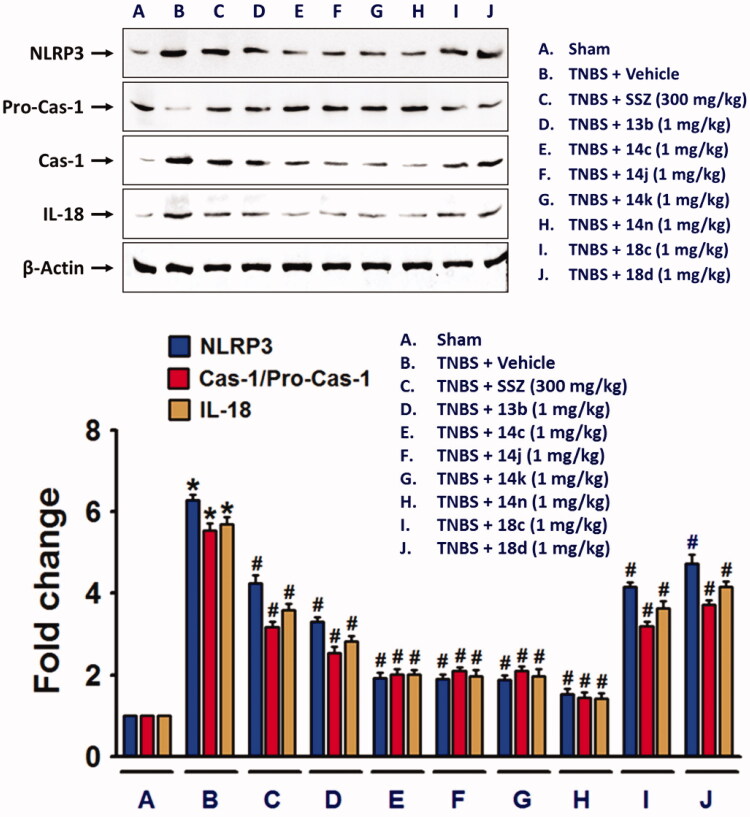
NLRP3 inflammasome formation in TNBS-treated rat colon was suppressed by the compounds. Total protein extracts from colon tissue homogenates were used for detection of NLRP3 inflammasome components. The bar graphs represent the mean ± SEM of protein expression quantified in three independent experiments. *p < 0.05, compared with vehicle-treated control group. #*p* < 0.05, compared with the TNBS-treated group.

Next, we also examined whether NLRP3 inflammasome, which plays an important role in IL-1β production^26^, was suppressed by the compounds. TNBS-induced alteration of NLRP3 inflammasome components, NLRP3, pro-caspase-1^27^, and cleaved and active caspase-1, were blocked by compounds, **13b**, **14c**, **14j**, **14k**, **14n**, **18c**, or **18d** (Figure 5). Similar to the effects of the compounds on cytokine expressions, **14n** exerted the strongest effect, which was followed by **14c**, **14j**, **14k**, **13b, 18c**, and **18d**. Consistent to the result of NLRP3 inflammasome activation, IL-18 level was significantly increased in the colon of TNBS-treated rats, and compounds, **13b**, **14c**, **14j**, **14k**, **14n**, **18c**, and **18d** significantly inhibited the TNBS-induced IL-18 expression.

**Figure 6.**
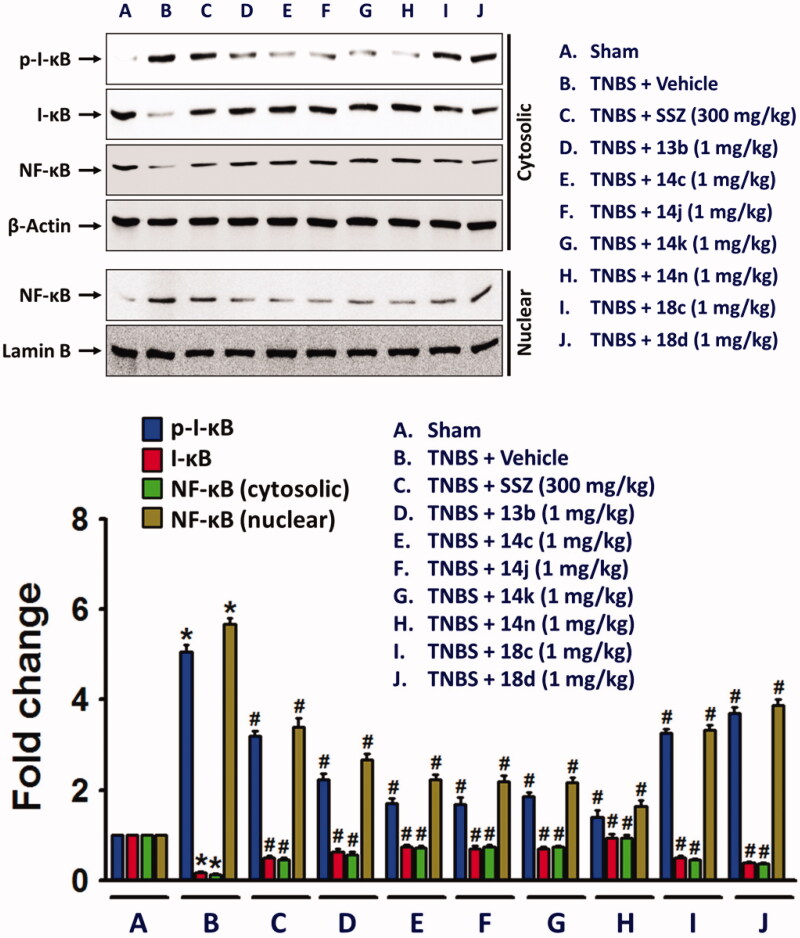
Inhibitory effects of the compounds on the nuclear translocation of NF-κB in along with increase in cytosolic phospho-I-κB. Cytosolic and nuclear proteins extracted from colon tissue homogenates were used for detection of phospho-I-κB, I-κB, and NF-κB. The bar graphs represent the mean ± SEM of protein expression quantified in three independent experiments. *p < 0.05, compared with vehicle-treated control group. #*p* < 0.05, compared with the TNBS-treated group.

In the colon of TNBS-treated rats, nuclear factor-kappa B (NF-κB), a transcription factor associated with expression of inflammatory cytokines and NLRP3 inflammasome was increased, while cytosolic NF-κB level was decreased in along with inhibitory-κB (I-κB) phosphorylation. However, the compounds significantly suppressed the nuclear translocation of NF-κB (Figure 6).

## 4. Discussion

TNF-α is a critical player in the pathogenesis of IBD, which is supported by the most efficacious effects of anti-TNF-α biologics in the clinic^28^. Based on the notion, we examined anti-IBD activity of various compounds against TNF-α-induced monocyte adhesion to colonic epithelial cells, an *in vitro* model of initial phase of colitis. In the previous reports, we found that 6-amino-2,4,5-trimethylpyridin-3-ol scaffold was effective against TNF-α-induced cell adhesion between monocyte and colon epithelial cells and against severe inflammation of colon induced by TNBS. Among the 6-aminopyridin-3-ols prepared, compounds **A **−** C** showed excellent efficacy in *in vivo* experiments^18^^,^^19^. 
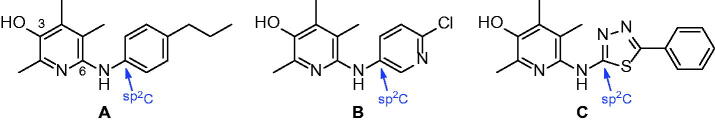


Structurally speaking, these strong analogues (**A **−** C**) contain some sort of sp^2^-carbon (indicated with arrows) attached to C(6)-nitrogen. In this study, we decided to further explore the chemical space of the substituents on C(6)-position. First, we wondered if nitrogen is essential on C(6)-position for activity and decided to replace it with another heteroatom, oxygen. The resulting alkoxy analogues (**8a**–**8f**) showed low to mediocre range activity in our *in vitro* assay and it seems that nitrogen is quite necessary for the position. Therefore, the next approach we decided to conduct based on the activity of the alkoxy-analogues is to keep nitrogen on C(6)-position and instead introduce carbonyl-like functional groups attached to C(6)-nitrogen as an alteration of sp^2^-carbon shown in previous papers. This type of analogues include ureido (**11**), thioureido (**12**), carbamato (**16**) and sulfonamido (**18**) analogues. As a result, many compounds shown in this paper were found to have very strong activity. *In vitro*, some compounds including thiourea and sulphonamide analogues showed up to 93% inhibition against TNF-α-induced cell adhesion at 1 μM concentration. It is the highest *in vitro* inhibitory activity measured with pyridin-3-ol analogues we synthesised so far. The order of the compounds having excellent *in vitro* effects was **14n**, **14k**, **14c**, **14j**, **13b**, **18c**, and **18d**. Such *in vitro* activity of the compounds was consistent with *in vivo* anti-colitis efficacy (1 mg/kg) on colitis signs and biochemical inflammatory markers, which was much better than SSZ (300 mg/kg). Among high activity compounds, **14n**, a thiourea analogue, showed the best *in vivo* activity represented by macroscopic parameters such as almost complete recovery of colon and body weights (95% and 99%, respectively) in TNBS-induced colitis rat model.

Although the exact aetiology of IBD has not yet been clarified, a defective innate immune response seems to play a critical role in the chronic gut inflammation^29^. Our current study showing an increase in the expressions of TNF-α, IL-6, and IL-1β, referred to as troika of pro-inflammatory cytokines in colon tissues of TNBS-treated rats, was consistent with many previous studies in IBD patients as well as animal models of IBD^30^^,^^31^. TNBS treatment also significantly increased colonic levels of IL-10 and TGF-β, known as anti-inflammatory cytokines. Although the expression level of IL-10 in experimental colitis is contradictory^32^, IL-10 level may be related to the inflammatory phase, increased at the initial phase and returned to baseline level in the resolution phase of inflammation^33^^,^^34^. Oral treatments with the compounds significantly suppressed TNBS-induced expressions of IL-10, in addition to TNF-α, IL-6, and IL-1β in colon tissues, indicating that an anti-colitis activity of the compounds led to the resolution phase of TNBS-induced inflammation. In the case of TGF-β, its production is upregulated by bacteria, viruses, cytokines, and dying cells, in an autocrine or paracrine manner^35^^,^^36^. TGF-β suppresses inflammatory responses similar to IL-10, although previous reports on TGF-β expression in IBD have shown contradictory results depending on active or inactive IBD^37^^,^^38^, TGF-β levels are elevated in inflamed area of gut tissues in IBD^39^. Consistently, our current results also showed that TNBS induced TGF-β increase in the colon, and oral administration of the compounds significantly reduced the level of TGF-β with the best effects of **14n**, **14k**, **14c**, and **14j**.

The innate immune system is activated by the pattern recognition receptors (PRRs) which recognise pathogen-associated molecular patterns (PAMPs) or endogenous danger-associated molecular patterns (DAMPs) generated during cellular injury or tissue damage^40–42^. PRRs comprise the membrane-bound toll-like receptors (TLRs) and the cytosolic sensory protein complexes such as NOD-like receptors (NLR)^43^. NLRP3, an NLR family member, has been highlighted as a contributing factor in the pathogenesis of IBD^44^. NLRP3 mediates the assembly and activation of the inflammasome, a multi-protein complex, which recognises cellular stresses and responds to them by activating caspase-1. Activated caspase-1 converts pro-IL-1β and pro-IL-18 into their active forms, IL-1β and IL-18, and induces pyroptosis, an inflammatory form of cell death^45^. In the present study, a significant increase in NLRP3 and caspase-1 in colon tissues by TNBS treatment are correlated to a high-level increase in IL-1β and IL-18. In addition, the correlation between casapase-1 increase and the significant reduction in junction molecules, E-cadherin, claudins, and ZO-1 in TNBS-treated colon implicates TNBS-induced mucosal damage through pyroptosis. Inhibitory effects of compounds, **14n**, **14k**, **14c**, **14j**, **13 b**, **18c**, and **18d** on TNBS-induced changes in inflammatory cytokine expression, casapse-1 and junction molecules suggested that anti-colitis effect of the compounds was associated with both anti-inflammatory and mucosal healing activities.

## 5. Conclusion

In summary, synthetic pyridinols **14n**, **14k**, **14c**, **14j**, **13b**, **18c**, and **18d** compounds showed strong inhibitory effects against TNF-α action *in vitro* and TNBS-induced colitis *in vivo*. The compound **14n**, which showed the highest inhibitory activity against colitis, may be a promising lead compound to develop anti-IBD therapeutics.
